# TNF-****α**** and IFN-s-Dependent Muscle Decay Is Linked to NF-**κ**B- and STAT-1****α****-Stimulated *Atrogin1* and *MuRF1* Genes in C2C12 Myotubes

**DOI:** 10.1155/2013/171437

**Published:** 2013-12-17

**Authors:** Barbara Pijet, Maja Pijet, Anna Litwiniuk, Małgorzata Gajewska, Beata Pająk, Arkadiusz Orzechowski

**Affiliations:** ^1^Department of Physiological Sciences, Faculty of Veterinary Medicine, Warsaw University of Life Sciences—SGGW, Nowoursynowska 159, 02-776 Warsaw, Poland; ^2^Electron Microscopy Platform, Mossakowski Medical Research Centre, Polish Academy of Sciences, Pawińskiego 5, 02-106 Warsaw, Poland

## Abstract

TNF-**α** was shown to stimulate mitogenicity in C2C12 myoblasts. Selected cytokines TNF-**α**, IFN**α**, or IFN**γ** reduced the expression of myosin heavy chain (MyHC IIa) when given together. Molecular mechanisms of cytokine activities were controlled by NF-**κ**B and JAK/STAT signaling pathways, as metabolic inhibitors, curcumin and AG490, inhibited some of TNF-**α** and IFN**α**/IFN**γ** effects. Insulin was hardly antagonistic to TNF-**α**- and IFN**α**/IFN**γ**-dependent decrease in MyHC IIa protein expression. Cytokines used individually or together also repressed myogenesis of C2C12 cells. Moreover, TNF-**α**- and IFN**α**/IFN**γ**-dependent effects on C2C12 myotubes were associated with increased activity of *Atrogin1* and *MuRF1* genes, which code ubiquitin ligases. *MyHC IIa* gene activity was unaltered by cytokines. Inhibition of NF-**κ**B or JAK/STAT with specific metabolic inhibitors decreased activity of *Atrogin1* and *MuRF1* but not *MyHC IIa* gene. Overall, these results suggest cooperation between cytokines in the reduction of MyHC IIa protein expression level via NF-**κ**B/JAK/STAT signaling pathways and activation of *Atrogin1* and *MuRF1* genes as their molecular targets. Insulin cotreatment or pretreatment does not protect against muscle decay induced by examined proinflammatory cytokines.

## 1. Introduction

Cachexia, an unintentional loss of lean body weight despite adequate nutrition, is a fatal complication of many diseases (cancer, congestive heart failure, diabetes, kidney failure, chronic obstructive pulmonary disease, rheumatoid arthritis, and HIV/AIDS), while the primary causal molecular mechanisms still remain unknown [1, 2]. Actually, it seems obvious that all these complications have something in common despite distinct etiology. Among several hypotheses, the insufficient anabolic action of insulin in response to rise in some cytokines during systemic inflammation attracted our attention. The loss of muscle tissue observed in elevated levels of proinflammatory cytokines seems to be linked to accelerated proteolysis rather than impaired protein synthesis [3]. Some authors [4] observed synergism between TNF-*α* and IFN-*γ* effects through NF-*κ*B activation. Moreover, Guttridge et al. [5] demonstrated that inhibition of myogenesis is NF-*κ*B dependent, as cytokine activation is responsible for the reduction of MyoD protein that controls muscle development [6]. Furthermore, as reported by Wheeler et al. [7], MyoD binding to myosin heavy chain IIb promoter region is necessary for myosin expression in fast twitch muscles. Interestingly, IGF-1 could not stop the effect of proinflammatory cytokines in myotube atrophy, even though it was shown to repress *Atrogin1* gene [8]. Nowadays, efforts to fight cachexia are based on targeting genes prior to their effects evoked in target organs [9]. It is believed that accelerated loss of skeletal muscle fibers and proteins which occur in muscle atrophy, muscle cachexia, and sarcopenia are driven by intrinsic mechanisms of autophagy [10], apoptosis [11], and decreased satellite cell activation [12]. Furthermore, the imbalance in regulation of skeletal muscle protein accretion leads to excessive activity of proteasome, cathepsins, calpains, and/or caspase proteolytic systems [3]. A great deal of papers referring to muscle cachexia points to erroneous activity of signaling pathways triggered by certain cytokines, such as IL-6, IL-1, TNF-*α*, IFN*γ*, and myostatin [13–17], respectively. TNF-*α* acting through TNFR1 is known to trigger two functionally opposite and sequential signals: (i) first to support cell viability through NF-*κ*B-dependent gene regulation; (ii) second to initiate intrinsic apoptosis with procaspase 8 activation [18]. Interestingly, NF-*κ*B transcription factor also plays a critical role in muscle cachexia [19–21]. Thus, deciphering the molecular mechanism of muscle cachexia would provide theoretical background for therapeutic use of some of the metabolic inhibitors. In this study murine C2C12 myotubes were subjected to short- and long term incubations with TNF-*α*, IFN*α*, IFN*γ*, and/or insulin as potent functional antagonists. The set-up of experiments was designed to verify the hypothesis that individually some cytokines could affect certain genes and proteins associated with muscle wasting, whereas together they add or synergize in their respective actions. It was assumed that they compete for the common transduction target (STAT-1*α*) that blocks internal signal essential for accelerated protein loss (NF-*κ*B). In this paper the results of both short (minutes) and long term (hours) studies performed on C2C12 myoblasts and myotubes are presented. We demonstrated that NF-*κ*B and JAK/STAT signaling pathways are intersected and that they control muscle growth and decay. IFN*α* reduced the level of STAT-1*α* protein linked to TRADD protein to release NF-*κ*B from STAT-1*α*-dependent inhibition. In differentiating C2C12 myotubes, TNF-*α* administration augmented cell growth, whereas it inhibited MyHC IIa protein expression in differentiated myotubes. To our surprise, TNF-*α*-induced effect was associated with both activation of *Myhc IIa*, *Atrogin1 *(also known as *MAFbx*), and *MuRF1* genes, but proteolysis took over protein accretion.

## 2. Materials and Methods

### 2.1. Materials

Media (Dulbecco's modified Eagles medium (DMEM) with Glutamax), PBS (including Ca^2+^ and Mg^2+^), antibiotics, and heat inactivated sera (fetal bovine serum—FBS and horse serum—HS) were purchased from Gibco Life Technologies (Grand Island, NY, USA). Mouse tumor necrosis factor alpha (TNF-*α*), interferon alpha (IFN*α*), interferon gamma (IFN*γ*), and insulin porcine (Sigma-Aldrich Chemical Co., St. Louis, MO, USA) were dissolved according to manufacturer's recommendations and kept frozen at −20°C. Metabolic inhibitors: curcumin (NF-*κ*B and proteasome inhibitor), LY294002 and PD98059 (PI3-K and MEK inhibitor, resp., Sigma-Aldrich Chemical Co., St. Louis, MO, USA), and tyrphostin (AG490, JAK inhibitor, Merck, Darmstadt, Germany) were dissolved in DMSO and depending on the type were kept frozen at −20°C or refrigerated at 4–8°C. All other reagents were cell culture tested, of high purity, and unless otherwise stated they were purchased from Sigma-Aldrich Chemical Co. (St. Louis, MO, USA). Plastics were from Becton Dickinson (BD Biosciences, Franklin Lakes, NJ, USA) and tubes for deep freezing were from Nunclon (Nunc, Roskilde, Denmark), while syringe filters were purchased from Corning-Costar Inc. (Cambridge, MA, USA).

#### 2.1.1. Muscle and Myotube Cell Cultures and Treatments

The mouse C2C12 cell line [22] was obtained from the European Collection of Animal Cell Cultures (ECAAC). Cells were initially suspended in growth media (GM) containing DMEM with Glutamax supplemented with 10% (v/v) fetal bovine serum (FBS), pen:strep (Penicillin:Streptomycin solution, 50 IU/mL/50 *μ*g/mL), Gentamicin sulfate 20 *μ*g/mL, Fungizone-Amphotericin B 1 *μ*g/mL, and plated onto plastic noncoated culture flasks or petri dishes. They were cultured at 37°C in a humidified 5% CO_2_ and 95% air in incubator. After reaching 70–80% confluence, myoblasts were subcultured by trypsynization and the same volume of cell suspension was seeded onto 100 mm petri dishes, 96-flatwell plates or multiwell 4 Chamber Culture Slides (Becton Dickinson, BD Biosciences, Franklin Lakes, NJ, USA) depending on the experimental protocol. For differentiating and differentiation states when the myoblasts reached 80% confluence, growth media were switched to differentiation media (DM) containing DMEM with Glutamax supplemented with 2% (v/v) horse serum (HS) and the same antibiotic:antimycotic mixture. During acute and chronic study of myogenic differentiation, DM was replaced by freshly prepared media containing 2% BSA (w/v) with or without experimental factors. In the case of mitogenicity study, GM in nonconfluent cells was directly replaced by 2% BSA (w/v)/DMEM with or without experimental factors. When the experimental factors were dissolved in DMSO, the equivalent volume of vehicle (0.1% v/v) was added to the control cells. DMSO-dissolved reagents were added exactly 30 min prior to the application of water-soluble reagents. Preliminary experiments were carried out with increasing concentrations of cytokines for different time points in order to choose the best time/concentration combination to adopt in our short- and long term studies. Differences in phosphorylation status and activities of particular proteins (NF-*κ*B, STAT-1*α*) being fast and transient phenomena were carried out as short term studies. Viability, mitogenicity, or changes in the protein expression were investigated in the long term study. There were dose- and time-dependent cell responses (Supplementary data 1, see Supplementary Materials available online at http://dx.doi.org/10.1155/2013/171437) suggesting that the best concentration setting in our experimental model (determined by the complete absence of cellular toxicity in MTT assay) was obtained using 10 ng/mL of TNF-*α*, IFN*α*, IFN*γ*, and 10 nM of insulin. Cells were removed from the culture plates using trypsin (harvesting), centrifuged in GM at 1000 rpm for 5 min, media were aspirated off, and cell pellets were resuspended in GM. For the differentiating state, after 24 h in GM, cells were washed with PBS and then incubated in DM for 3 to 5 days. Media were changed every day. For differentiation state, the cells were allowed to incubate in DM for 6 days prior to treatment with cytokines or inhibitors carried out for another 2 days (8 days altogether). Media were changed every other day. Myotubes were harvested on Day 8 of differentiation (i.e., 6 days without treatment + 2 days with treatment). Floating dead cells were removed during media change or washing with PBS and were not included in these experiments.

#### 2.1.2. Determination of Cell Viability and Mitogenicity

Cell viability was based on the ability of cells grown on 96-well plates to convert soluble MTT [3-(4,5-dimethylthiazol-2-yl)-2-5-diphenyltetrazolium bromide] into an insoluble purple formazan reaction product with minor modifications to protocol described by Jacobson et al. [23]. Briefly, cells were uniformly seeded in 96-well flat bottomed plates and grown in GM for 24 h. Confluent cultures were washed with PBS and then exposed to DM including (or not) experimental factor(s) for 5 successive days. Media were changed every day and relative viability (percent of control) was evaluated at each day (1, 2, 3, 4, 5). Media were removed, cells were washed three times with PBS, and were further incubated with MTT for 1 h at 37°C in a humidified 5% CO_2_ and 95% air in incubator. Next, MTT solution was removed and water insoluble formazan was immediately dissolved in DMSO.

Cell mitogenicity was determined by crystal violet (CV) assay on identical 96-well flat bottomed multiwell plates. Cells were uniformly seeded and grown in GM for 24 h. Mitogenicity was measured on day 3 of culture. Initially, cells were kept in GM for 24 h, followed by 24 h incubation with the experimental factor(s) dissolved in serum-free 2% BSA/DMEM, and finally recovered in GM for another 24 h. Cells were exposed (or not) to experimental factors during 24 h. Upon completing the experiment, cells were washed with PBS and fixed with two-step bath in ice-cold methanol (70% followed by 100%, v/v, 20 min, 4°C). Cells were immersed in 0.2%, w/v crystal violet solution in dd.H_2_O with ethanol 2% (v/v) for 10 min. Subsequently, they were gently washed with dd.H_2_O, air dried, and incubated with SDS solution (1%, w/v in dd.H_2_O). The absorbances for MTT and CV were measured at 490 and 570 nm, respectively, with ELISA reader type Infinite 200 (TECAN, Austria). Relative percentages (versus nontreated control) of viable or proliferating cells were measured by MTT conversion into purple formazan and quantity of CV bound to cellular DNA, respectively.

#### 2.1.3. Antibodies, Immunoblotting, Immunoprecipitation, Transcriptional Activity of STAT-1*α*, Immunofluorescence, and Microscopic Imaging

For whole-cell lysates the cells were cultured with or without experimental factors indicated in the legend of Figure 7(a), harvested, washed, and lysed with RIPA lysis buffer (1x PBS, 10 mL/l Igepal CA-630, 5 g/L sodium deoxycholate, 1 g/L SDS) supplemented with 0.4 mM PMSF; 10 *μ*g/mL of aprotinin and 10 *μ*g/mL of sodium orthovanadate were added. To lyse the cell pellets, cells were broken up by repetitive triturating with the syringe with attached needle (21 G, 0.8 mm diameter). Cell suspension was then left on ice (4°C) for 30 min and centrifuged for another 5 min (4°C, 10 000 g). Next, viscous solution was divided into smaller volumes and transferred to fresh Eppendorf tubes and stored at −80°C until used. For protein quantification in the whole-cell lysates, a protein-dye-binding method of Bradford [24] with commercial reagent was used (Bio-Rad Laboratories, Hercules, CA, USA). To separate cytoplasmic and nuclear fractions, cells were washed, and after centrifugation cell pellets were resuspended in 400 *μ*L of ice-cold buffer (10 mM HEPES pH 7.9; 10 mM KCl; 0.1 mM EDTA; 0.1 mM EGTA; 1 mM DTT; 0.5 mM PMSF) and incubated on ice for 15 min. Then 25 *μ*L of a 10% solution of Igepal CA-630 was added. After centrifugation, supernatants containing cytoplasm were transferred to fresh tubes and were stored at −80°C. Nuclear pellets were resuspended in 200 *μ*L RIPA buffer (1x PBS; 1% Igepal CA-630; 0.5% sodium deoxycholate; 0.1% SDS; aprotinin (available as a liquid from Sigma-Aldrich Chemical Co.); 30 *μ*L added to 1 mL of buffer; 1 mM sodium orthovanadate) and were passed through a 21-gauge needle. PMSF (0.4 mM) was added and cells were incubated 30 min on ice. After centrifugation, cytoplasmic and nuclear lysates were stored at −80°C until analysis.

Cell lysates (equal protein loads of 50 *μ*g) were subjected to SDS-PAGE (10–12.5% of gel, depending on the MW of protein) at 150 V, next they were transferred at 100 V for 2 h to polyvinylidene difluoride (PVDF) membrane and immunoblotted with antibodies against phosphorylated and/or nonphosphorylated forms of proteins: actin, NF-*κ*B (p65), I*κ*B*α*, STAT1-*α*, P(Tyr701)-STAT1-*α*, MyoD, and myogenin (Santa Cruz Biotechnology, Santa Cruz, CA, USA) or MyHC IIa (Sigma Aldrich Chemical Co., St. Louis, MO, USA). Working antibody concentrations (from 1 : 200 to 1 : 1000) varied depending on the protein detected and were applied according to the manufacturer's recommendations. After detection with antibodies which distinguished phosphorylated proteins, the same blot was reprobed with antibodies against the nonphosphorylated form of the same protein in order to verify that equal amounts of protein were always loaded and to identify the proteins. In order to reprobe a blot with a different antibody, the membrane was incubated in stripping buffer (100 mM *β*-mercaptoethanol, 2% (w/v) SDS, 62.3 mM Tris, pH 6.7) for 30 min at 30°C, and then reblocked. Secondary polyclonal antibodies (Santa Cruz Biotechnology, Santa Cruz, CA, USA) raised against respective species and conjugated to horseradish peroxidase were used for detection, followed by enhanced chemiluminescence assay (Amersham International, Aylesbury, U.K.). After exposure and processing, the films were scanned and analyzed using Kodak EDAS 290/Kodak 1D 3.5 system.

For immunoprecipitation at particular time points the cells were scraped from substratum in 0.5 mL RIPA buffer, and after repetitive triturating with the 21 gauge needle, 0.4 mM PMSF was added and cells were incubated 30 min on ice. After centrifugation the protein concentration was determined by a protein-dye-binding method as previously described. Cell lysates containing 900 *μ*g of protein were incubated overnight at 4°C with 1.5 *μ*g rabbit polyclonal anti-STAT-1*α* IgG and for an additional 3 h were incubated with 30 *μ*L protein A/G bead slurry (Santa Cruz, CA). Beads were washed 4 times with ice-cold RIPA buffer, boiled with sample buffer (2x Laemmli buffer, Sigma-Aldrich Chemical Co., St. Louis, MO, USA), and separated by 10% SDS/PAGE. After electrotransfer, the membranes were immunostained for TRADD protein by standard Western blot procedure.

NF-*κ*B and STAT-1*α* transcriptional activities were quantified with TransAM Kits (Rixensart, Belgium). These are sensitive, nonradioactive transcription factor ELISA Kits that facilitate the study of transcription factor activation in mammalian tissue and cell extracts. The active form of STAT-1*α* contained in nuclear extracts was specifically bound to the immobilized oligonucleotide containing STAT consensus binding site (5′-TTCCCGGAA-3′). The primary antibody used to detect STAT recognized only the alpha subunit of STAT-1*α*, that is, accessible only when STAT-1*α* was activated and bound to its target DNA. Similarly, TransAM NF-*κ*B Kit contained a 96-well plate on which oligonucleotide containing the NF-*κ*B consensus site (5′-GGGACTTTCC-3′) has been immobilized. The active form of NF-*κ*B contained in nuclear or whole-cell extract specifically binds to this oligonucleotide. The primary antibodies used to detect NF-*κ*B recognized an epitope on p65 or p50, that is, accessible only when NF-*κ*B is activated and bound to its target DNA. Finally, an HRP-conjugated secondary antibody provided a sensitive colorimetric readout that was quantified by spectrophotometry (450 nm).

For immunofluorescence, C2C12 myoblasts were propagated in multiwell 4 chamber culture slides and treated (or untreated) at appropriate times (Supplementary data 2) with experimental factors dissolved in 20 g/L BSA/DMEM or 0.1% v/v DMSO in 20 g/L BSA/DMEM. After the experiment ended, the cells were fixed as follows: washed twice with PBS, fixed in 3.7% (v/v) formaldehyde for 15 minutes in room temperature, washed twice with PBS containing 10 g/L BSA/PBS, and incubated for 10 min in RT in Triton X-100 solution (0.5% v/v in PBS). Next, cells were washed twice with PBS. Prior to staining, the cells were washed three times with 10 g/L BSA/PBS, and then incubated overnight at 4°C with primary rabbit polyclonal anti-NF-*κ*B diluted 1 : 100. After incubation the cells were washed three times with 10 g/L BSA/PBS and subsequently incubated for 1 hour in dark at RT with secondary chicken anti-rabbit antibody conjugated to Alexa Fluor 488 (Molecular Probes Inc., Eugene, OR, USA) diluted 1 : 500. The cells were then washed three times with PBS and incubated with 7-aminoactinomycin D (7′-AAD) water solution for 15 min at RT to visualize the cell nuclei. Afterwards, the cells were washed five times with 10 g/L BSA/PBS, the chamber walls were removed, and coverslips were mounted on microscope slides using an antifade Fluoromount (Sigma Aldrich Chemical Co., St. Louis, MO, USA). As a negative control, only secondary antibodies were used. Cells were visualized using confocal microscope FV-500 (Olympus Optical Co., Hamburg, Germany). The fluorescence excitation was provided by 488 nm and 543 nm He-Ne laser beams. Fluorescence was measured using dichroic mirrors and filters for 505, 525 nm, 560, and 610 nm wavelengths. Acquired data were stored in a series of 12 bit grey images separately and colored artificially by software.

Morphological changes and cell survival were monitored under an inverted phase-contrast microscope (Olympus CK40, model: ICD703WP). The formation of myotubes was monitored by obtaining photomicrographs using digital camera (CCD Color Camera, Hamburg, Germany).

#### 2.1.4. RNA Isolation and Quantitative Real-Time Reverse-Transcription-Polymerase Chain Reaction (qRT-PCR)

Total RNA was extracted from myotubes with the Total RNA Maxi Kit (A&A Biotechnology, Gdynia, Poland), according to manufacturer's protocol. RNA was frozen at −76°C before the performing the reverse transcription reaction (RT-PCR). Then, 500 *μ*g of total RNA was purified on silica gel and reverse-transcribed with Enhanced Avian HS RT-PCR-100 Kit (Sigma-Aldrich, Taufkirchen, Germany). Reaction mixture was based on anchored Oligo(dT)_23_ (0.5 *μ*g/*μ*L in water for PCR). RT-PCR was carried out using Mastercycler Personal (Eppendorf, New York, NY, USA). First, the RNA samples (200 ng/mL) were incubated for 10 min at 70°C. Then water for PCR, 10XAMV-RT buffer [500 mM Tris-HCl, pH 8.3, 400 mM KCl, 80 mM MgCl_2_, 10 mM DTT, RNAse inhibitor (20 U/*μ*L), and reverse transcriptase AMV (20 U/*μ*L w 200 mM KH_2_PO_4_, pH 7.2, 2 mM DTT, 0.2% (v/v) triton, 50% (v/v) glycerol] were added at 4°C. The samples were subjected to RT-PCR for 50 min at 48°C. After completing the reaction the concentration of newly synthesized cDNA was measured in the NanoDrop 1000 (NanoDrop Technologies, Wilmington, NC, USA) at *λ* = 230 nm. cDNA was kept frozen at −76°C until further analyses. To perform real time PCR reaction, cDNA was combined with 25 *μ*M of each primer (sense and antisense) and SYBR green (LightCycler FastStart DNA Master SYBR Green I and LightCycler Control Kit DNA; Roche Diagnostics, Warsaw, Poland). The qRT PCR measurements of individual cDNAs were performed in triplicates using SYBR green dye to measure duplex DNA formation with the LightCycler (Roche Diagnostics, Warsaw, Poland). The results were analyzed with LightCycler3 Front Screen. *18S rRNA* was used as reference gene. The sequences of the primers sets used are shown in the attached Table 1 (GenBank). The relative mRNA levels of the target genes were determined using the relative standard curve.

PCR conditions were as follows: denaturation for each cycle 95°C (35 cycles, 10 sec for each cycle); annealing both for *Myhc IIa *and* Atrogin1* 60°C (35 cycles, 0–10 sec for each cycle); for *MuRF1* 58°C (35 cycles, 0–10 sec for each cycle); for *18S rRNA* 56°C (35 cycles, 0–10 sec for each cycle); and elongation 72°C (35 cycles, 4-5 sec for each cycle).

### 2.2. Statistical Analysis

Each experiment was repeated at least three times. The data are expressed as the means ± SE. Statistical analyses were performed using one-way analysis of variance (ANOVA) followed by Tukey's, Newman-Keuls'a or Benferroni multiple range test. If necessary, the selection of particular posthoc test (Newman-Keuls, Tukey, or Benferroni) was performed after the same critical difference for the first comparison was tested. Regression analysis was carried out to draw appropriate dose-response or time-course curves. *P* values of less than 0.05 were considered statistically significant. Statistical differences from control cells were indicated by asterisks (*P < 0.05; **P < 0.01; ***P < 0.001), whereas statistical differences between the treatments and untreated control cells were ticked with different lower case letters (bar charts). Statistical analyses were performed using GraphPad Prism version 5.0 software (GraphPad Software Inc., San Diego, CA, USA).

## 3. Results

### 3.1. TNF-*α* Stimulates Both Viability and Mitogenicity of C2C12 Myoblasts in NF-*κ*B- and JAK/STAT-Dependent Manner

**Figure 1:**
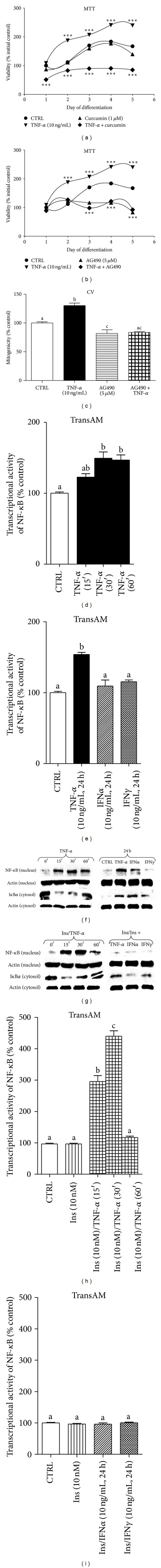
Short- and long term effects of TNF-*α*, IFN*α*, IFN*γ* (10 ng/mL each), insulin (10 nM), and metabolic inhibitors (at concentrations indicated) upon cell viability during 5 subsequent days of myogenic differentiation (a, b), cell mitogenicity determined at the 3rd day of experiment (c), NF-*κ*B transcriptional activity in the 3rd day of myogenesis (d, e, h, i), cytosolic I*κ*B and nuclear NF-*κ*B expression levels upon TNF-*α*, and additional insulin pretreatment in the 3rd day of myogenesis (f, g). Actin was used as loading control. The results are indicative of three independent experiments.

Addition of TNF-*α* (10 ng/mL) to the medium stimulated viability (by 88 ± 2.82 to 140% ± 6.84) and mitogenicity (by 30% ± 4.24) of differentiating C2C12 myoblasts (Figures 1(a) and 1(c), *P* < 0.001 compared to initial control). AG490 (5 *μ*M) used individually impaired cell viability at days 3–5 of differentiation (Figures 1(a) and 1(b), *P* < 0.001 compared to initial control), but curcumin (1 *μ*M) did not exert this effect. The effects of TNF-*α* on cell viability and mitogenicity were abolished by curcumin (NF-*κ*B inhibitor) and AG490 (JAK inhibitor) administration (Figures 1(b) and 1(c)).

### 3.2. TNF-*α* Stimulates the Expression and Transcriptional Activity of NF-*κ*B in C2C12 Myotubes

To verify the intracellular level of NF-*κ*B (p65) in 3-day-old C2C12 myotubes, nuclear fractions were isolated and immunoblotted, whereas transcriptional activity was evaluated using TransAM method. Nuclear expression of NF-*κ*B protein increased as early as 15 minutes after TNF-*α* administration (Figure 1(f)). There was a marked drop in cytoplasmic I*κ*B at the same time (Figure 1(f)). As shown on Figure 1(d), when C2C12 myotubes were exposed to TNF-*α* (10 ng/mL), the transcriptional activity of NF-*κ*B was significantly elevated in 30th (by 49% + 8.9) and 60th minute (by 47% ± 7.15) of treatment (*P* < 0.01 compared to control). Furthermore, also after 24 hours of TNF-*α* administration both NF-*κ*B expression and its activity were still significantly higher (by 54% ± 2.89, Figures 1(e)-1(f), *P* < 0.01 compared to control). In contrast to short term treatment (minutes), after 24 hours of TNF-*α* administration the expression level of I*κ*B*α* protein was increased in the cytoplasmic fraction (Figure 1(f)). Neither IFN*α* nor IFN*γ* affected the activity of NF-*κ*B in C2C12 myotubes in the long term study (Figure 1(e), *P* > 0.05 compared to control).

### 3.3. Pretreatment of C2C12 Myoblasts with Insulin Raises TNF-*α*-Dependent Activity of NF-*κ*B, Though Insulin Does Not Alter NF-*κ*B Response in C2C12 Myotubes regardless of Insulin Pretreatment and Cotreatment with TNF-*α*, IFN*α*, or IFN*γ*

Pretreatment with insulin (72 h, 10 nM) prior to TNF-*α* (10 ng/mL) administration significantly increased TNF-*α*-dependent NF-*κ*B activity in 15th, 30th but 60th minute (by 3-fold in the 15th and by 4.5-fold in the 30th minute of TNF-*α* posttreatment, Figure 1(h), *P* < 0.001 compared to control) in 3-day old C2C12 myotubes. In contrast, insulin pretreatment neither changed NF-*κ*B activity (Figure 1(i), *P* > 0.05) nor expression when administered as pretreatment and cotreatment with IFN*α* or IFN*γ* (Figure 1(g)).

### 3.4. Pretreatment with IFN*α* or IFN*γ* Does Not Affect TNF-*α*-Induced Nuclear Expression of NF-*κ*B Though It Increases Its Activity in C2C12 Myotubes: Insulin Could Not Block TNF-*α*-Induced Nuclear Expression and Increased Activity of NF-*κ*B Potentiated by Interferons

**Figure 2:**
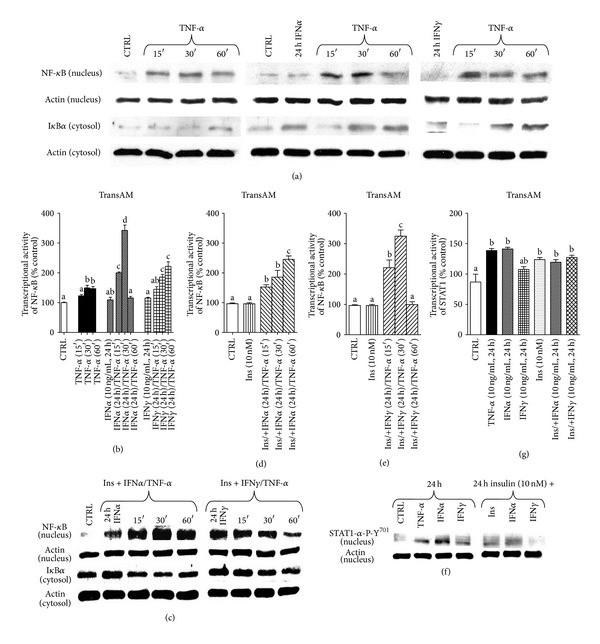
Short- and long term effects of TNF-*α*, IFN*α*, IFN*γ* (10 ng/mL each), insulin (10 nM), and metabolic inhibitors (at concentrations indicated) given alone, as pretreatment or cotreatment (with +) in the 3rd day of myogenesis. Changes in the cytosolic I*κ*B and nuclear NF-*κ*B expression levels upon TNF-*α* and additional IFN*α*, or IFN*γ* pretreatment (a), NF-*κ*B (b, d, e) and STAT-1*α* transcriptional activity (g), NF-*κ*B/I*κ*B cytosolic and nuclear protein expression levels upon TNF-*α* and additional insulin, IFN*α* or IFN*γ* pretreatments or co-treatments (with +) (c), and STAT-1*α*-P-Y^701^ nuclear protein expression levels upon TNF-*α*, IFN*α*, or IFN*γ* treatment or additional insulin pretreatment and co-treatment (with +) (f). Actin was used as loading control. The results are indicative of three independent experiments.

Preincubation with interferons (IFN*α*, IFN*γ*, 10 ng/mL) sensitized cellular responses to TNF-*α* administration in 3-day old C2C12 myotubes as evidenced by NF-*κ*B activity (IFN*α* pretreatment increased TNF-*α* response by 2-fold in the 15th and by 3.5-fold in the 30th minute, while IFN*γ* by 45% ± 9.08, 85% ± 9.18, and 122% ± 15.16 at 15th, 30th, and 60th minute of treatment, respectively, Figure 2(b), *P* < 0.001 compared to control). NF-*κ*B activity stimulated by TNF-*α* and potentiated by IFN*α* in 3-day-old C2C12 myotubes was additionally substantiated by nuclear NF-*κ*B setting observed in confocal microscopy (Supplementary data 2). Visualization confirmed that these reactions occurred, even though NF-*κ*B expression levels remained unchanged. When insulin was used together with interferons in one-day pretreatment, the effect of insulin on NF-*κ*B protein expression (Figure 2(c)) and activity in 3-day old C2C12 myotubes was also higher (insulin + IFN*α* pretreatment increased TNF-*α* response by 52% ± 7.64, 86% ± 22.63, and 145% ± 11.33 at 15th, 30th, and 60th minute, respectively, (Figure 2(d), *P* < 0.01), while insulin + IFN*γ* by 121% ± 25.19 and 225% ± 20.2 in the 15th and 30th minute of TNF-*α* treatment (Figure 2(e), *P* < 0.001 compared to control).

### 3.5. Pretreatment with TNF-*α* or IFN*α* but Not IFN*γ* Increased the Nuclear Protein Expression and Activity of STAT-1*α*-P-Y^701^ in C2C12 Myotubes: Insulin Does Not Significantly Affect the Effects of Cytokines on Nuclear Protein Expression and Activity of STAT-1*α*-P-Y^701^

Preincubation with cytokines (TNF-*α* or IFN*α*, 10 ng/mL, 24 h) increased STAT-1*α*-P-Y^701^-nuclear protein expression levels and activity in 3-day-old C2C12 myotubes by 39% ± 3.35 and 41% ± 2.78, respectively, (Figure 2(g), *P* < 0.01 compared to control). Insulin given together with IFN*α* did not reduce elevated STAT-1*α*-P-Y^701^ activity and nuclear protein expression level (Figures 2(g) and 6(b), *P* > 0.05 compared to control).

### 3.6. TNF-*α* Increased Nuclear Expression and Activity of STAT-1*α*-P-Y^701^ in C2C12 Myotubes: Insulin Pretreatment Hardly Stops the Effect of TNF-*α* on Nuclear Protein Expression Levels and Activity of STAT-1*α*-P-Y^701^

**Figure 3:**
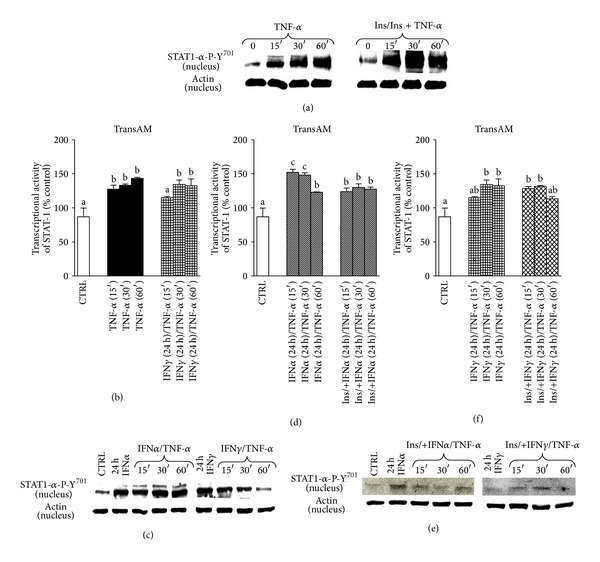
Short- and long term effects of TNF-*α*, IFN*α*, IFN*γ* (10 ng/mL each), and insulin (10 nM) given alone, as pretreatment or together in the 3rd day of myogenesis. STAT-1*α*-P- Y^701^ nuclear protein expression levels upon TNF-*α* and additional insulin pretreatment (a), STAT-1*α* transcriptional activity (b, d, f), Y^701^-phospho-STAT-1*α* nuclear protein expression levels upon TNF-*α* and additional IFN*α* or IFN*γ* pretreatment (c), STAT-1*α*-P- Y^701^ nuclear protein expression levels upon TNF-*α* and additional IFN*α* or IFN*γ* pretreatment, and subsequent insulin and IFN*α* or IFN*γ* cotreatment (e). Actin was used as loading control. The results are indicative of three independent experiments.

Similarly to NF-*κ*B, myotubes responded to TNF-*α* administration with considerable raise in nuclear protein expression levels and activity of STAT-1*α*-P-Y^701^ (increase by 28% ± 5.83, 33% ± 1.98, and 43.17 ± 1.67 at 15th, 30th, and 60th minute of treatment, respectively, Figure 3(a), *P* < 0.01 compared to control). Insulin pretreatment (10 nM, 72 h) hardly affected the cellular response to TNF-*α* with respect to STAT-1*α*-P-Y^701^ activity as it remained significantly higher than in control conditions during 15 (44% ± 6.24, *P* < 0.001), 30 (37.28% ± 4.54, *P* < 0.001), and 60 (17% ± 4.0, *P* < 0.05) minutes of treatment (Figures 3(d) and 3(f)).

### 3.7. Pretreatment with IFN*α* but Not IFN*γ* Assists TNF-*α* in Increased Nuclear Expression and Activity of STAT-1*α*-P-Y^701^ in C2C12 Myotubes: Insulin Does Not Overturn the Effect of IFN*α* on Nuclear Expression of STAT-1*α*-P-Y^701^

When 3-day-old C2C12 myotubes were pretreated with IFN*α* (10 ng/mL, 24 h) followed by TNF-*α* challenge, STAT-1*α*-P-Y^701^ protein expression was similar to the conditions in which TNF-*α* was given alone (Figure 3(c)). Additionally, IFN*α* markedly ameliorated STAT-1*α* activity by 52% ± 4.52, 48.12 ± 3.26, and 23% ± 0.47 at 15th, 30th, and 60th minute of treatment, respectively, (Figure 3(d), *P* < 0.001 versus control). When IFN*α* was replaced by IFN*γ* (10 ng/mL, 24 h), TNF-*α*-dependent rise in nuclear protein expression levels of STAT-1*α*-P-Y^701^ was halted (Figure 3(c)), whereas the activity of STAT-1*α*-P-Y^701^ did not differ from TNF-*α* administered alone (increase by 28% ± 8.83, 33% ± 1.98, 43.17 ± 1.67 at 15th, 30th, and 60th minute of TNF-*α* treatment, resp., Figure 3(f), *P* < 0.001 versus control). Additional preincubation with insulin (10 nM, 72 h) neither affected the response to IFN*γ* nor to IFN*α* pretreated cells in STAT-1*α*-P-Y^701^ expression levels and transcriptional activities (Figures 3(e) and 3(f)).

### 3.8. IFN*α* and IFN*γ* Facilitate TNF-*α*-Dependent Decrease of MyHC IIa Protein Expression Levels in C2C12 Myotubes. Insulin Does Not Prevent TNF-*α*-Dependent Loss of MyHC IIa

**Figure 4:**
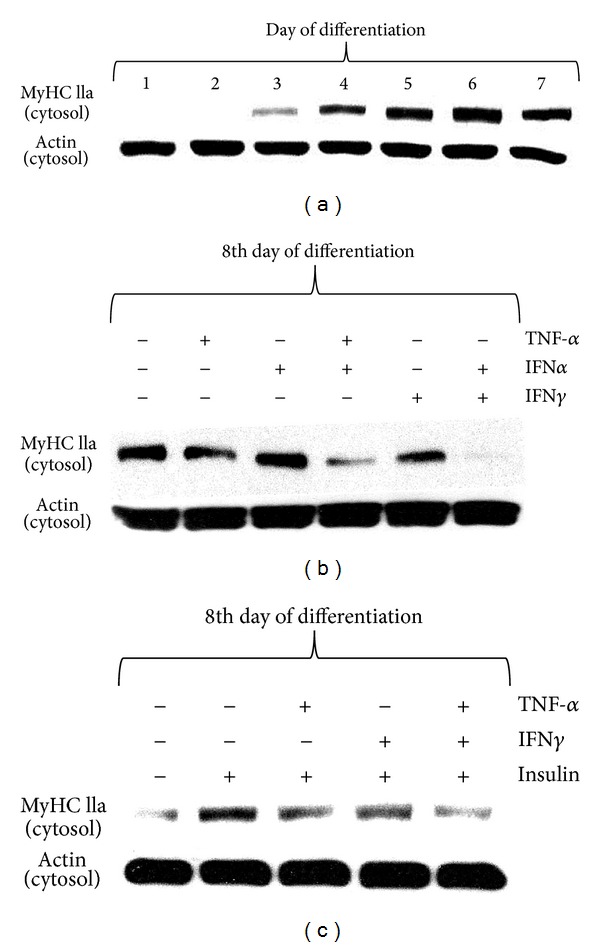
Changes in the cytosolic myosin heavy chain IIa (fast form) expression levels during 7 days of myogenesis from C2C12 myoblasts (a). Long term effects of TNF-*α*, IFN*α*, IFN*γ* (10 ng/mL each), or insulin (10 nM) given alone or together on the cytosolic myosin heavy chain IIa (fast form) expression levels in the 8th day of myogenesis (b, c). Actin was used as loading control. The results are indicative of three independent experiments.

MyHC IIa protein expression level has been selected as a marker of protein gain/loss in C2C12 myotubes. Its expression increased progressively during the consecutive days of differentiation (Figure 4(a)). Initially, the raise in MyHC IIa protein level was noted at the 3rd day of differentiation (extensive fusion of myoblasts), and then it gradually increased to the 6th day with no additional elevation at day 7 (Figure 4(a)). It was thought that at least 6 days of differentiation should be considered as the starting point for evaluation of changes in MyHC IIa protein expression. TNF-*α* (10 ng/mL, 48 h) was shown to inhibit the expression of MyHC IIa in C2C12 myotubes (8-day-old), while additional presence of IFN*α* or IFN*γ* (10 ng/mL, 48 h) amplified TNF-*α*-dependent drop in MyHC IIa protein expression (Figure 4(b)). Insulin pretreatment (10 nM, 24 h) was apparently negligible to the TNF-*α* effect induced in C2C12 myotubes (Figure 4(c)).

### 3.9. Elevated Expression of Nuclear NF-*κ*B Evoked by TNF-*α* Is Controlled by Proteasome and JAK Activity

**Figure 5:**
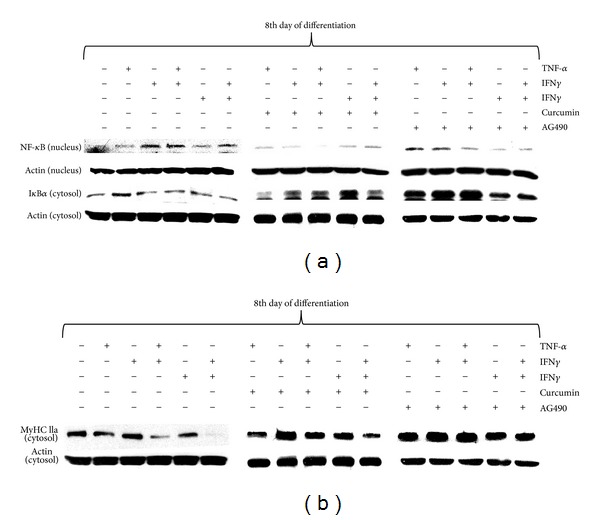
Changes in the cytosolic I*κ*B and nuclear NF-*κ*B expression levels in the 8th day of myogenesis (a). Long term effects of TNF-*α*, IFN*α*, IFN*γ* (10 ng/mL each), or metabolic inhibitors (curcumin 1 *μ*M, AG 490 5 *μ*M) given alone or together. Changes in the cytosolic myosin heavy chain IIa (fast form) expression levels in the 8th day of myogenesis (b). Actin was used as loading control. The results are indicative of three independent experiments.

The administration of either curcumin (1 *μ*M, proteasome inhibitor) or AG490 (5 *μ*M, JAK inhibitor) together with TNF-*α* and/or IFN*α* or IFN*γ* during 48 hours (from 6th to 8th day of differentiation) reversed the nuclear raise in NF-*κ*B protein expression to the control level (Figure 5(a)). Accordingly, as shown on Figure 5(a), both inhibitors were equally potent in elevating cytosolic I*κ*B expression levels. As both NF-*κ*B activity and nuclear expression levels were associated with concomitant drop in MyHC IIa protein expression, the above-mentioned inhibitors were also used to determine whether they can protect C2C12 myotubes from the TNF-*α*-dependent loss of MyHC IIa. In agreement with our suggestion, both inhibitors (curcumin and AG490) were capable to prevent the TNF-*α*-dependent loss of MyHC IIa expression levels in C2C12 myotubes, when compared with the control level (Figure 5(b)).

### 3.10. Decreased Expression of MyHC IIa Induced by TNF-*α* and IFN*α* or IFN*γ* in Differentiating C2C12 Myotubes Is Accompanied by Lower Expression of Selected MRFs (Muscle Regulatory Factors) and It Depends on NF-*κ*B and STAT Activity

**Figure 6:**
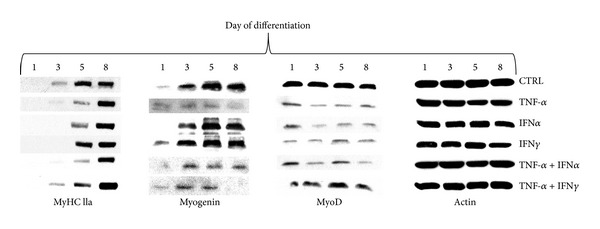
Changes in the cytosolic myosin heavy chain IIa (fast form), myogenin, and MyoD expression levels in selected days (1, 3, 5, 8) of 8 days of myogenesis from C2C12 myoblasts. Long term effects of TNF-*α*, IFN*α*, IFN*γ* (10 ng/mL each), or metabolic inhibitors (curcumin 1 *μ*M, AG 490 5 *μ*M) given alone or together. Actin was used as loading control. The results are indicative of three independent experiments.

As MRFs are critical for muscle differentiation and maturation of myotubes, two different transcription factors were chosen for the study, namely, MyoD known as primary muscle regulatory factor and myogenin secondary to MyoD. As presented in Figure 6 MyoD protein expression was reduced all along the treatment, irrespective to cytokine, whereas the level of myogenin protein was decreased by TNF-*α* treatment or cotreatment only. Additional administration of IFN*α* or IFN*γ* did not alter the myogenin response to TNF-*α* (Figure 6). However, the expression of MyHC IIa protein decreased accordingly. Both the number and diameter of myotubes were reduced after individual treatment with TNF-*α* or TNF-*α* and IFN*γ* but not with IFN*α*. They were restored to normal after simultaneous administration of curcumin or AG490 (data not shown).

### 3.11. Combined Treatments of TNF-*α* and IFN*α* or IFN*γ* Decrease the Affinity of STAT-1*α* to TRADD Protein in C2C12 Myotubes

**Figure 7:**
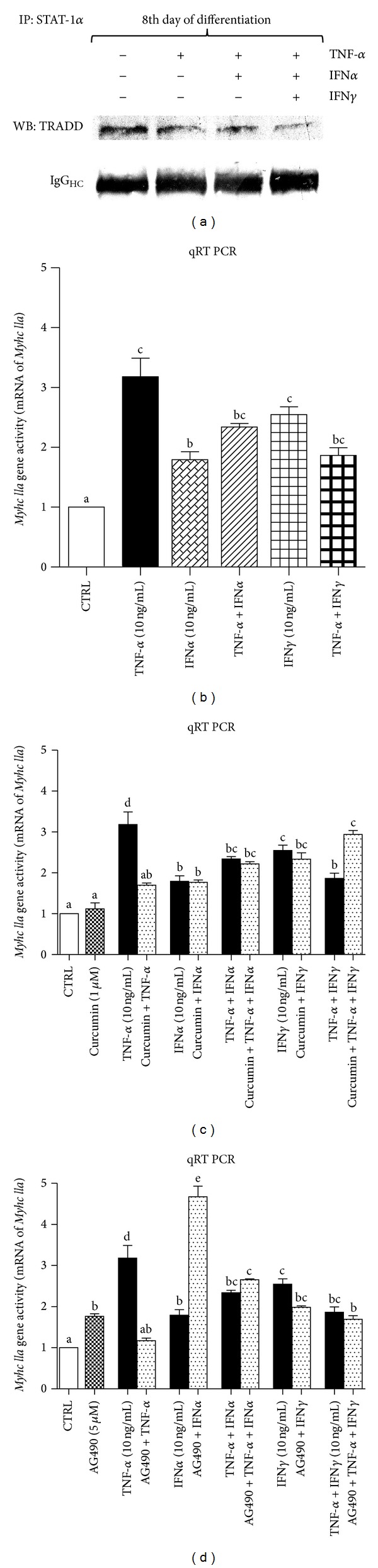
Immunoprecipitation of STAT-1*α* shows time-dependent loss of immunoreactive TRADD in the precipitates upon TNF-*α* and/or IFN*α* or IFN*γ* cotreatment in the 8th day of myogenesis (a). IgG was used as equal input control. The results are indicative of three independent experiments. Long term effects (eight days) of TNF-*α*, IFN*α*, and IFN (10 ng/mL each) given alone or together on the *Myhc IIa* gene activity (b). Long term effects (eight days) of TNF-*α*, IFN*α*, IFN*γ* (10 ng/mL each, filled bars), or metabolic inhibitors (curcumin 1 *μ*M) given alone (filled bars) or together (dotted bars) on *Myhc IIa* gene activity (c, d). Fold increase was calculated according to the formula described in Section 2.

In this study, it was assumed that STAT-1*α* protein is activated either by IFN*α* or IFN*γ* after binding to respective IFNAR or IFNGR. STAT-1*α* was previously reported to be bound to TRADD protein [41–44] and inhibits NF-*κ*B-dependent death signal. As STAT-1*α* protein is modulated by interferons, also NF-*κ*B signaling pathway can be released from inhibition when both TNF-*α* and IFN*α* or IFN*γ* are administered. To verify this assumption STAT-1*α* protein complexes were isolated by immunoprecipitation, and TRADD protein was immunoblotted after PAGE. As it is shown in Figure 7(a), the expression of TRADD that was bound to STAT-1*α* was the highest in untreated myotubes, and it was lowered by TNF-*α* and further cotreatment with IFN*α* and IFN*γ*.

### 3.12. TNF-*α*, IFN*α*, and IFN*γ* Stimulate *Myhc IIa* Gene Activity, and This Process Is Controlled by NF-*κ*B and STAT-1*α* as the Administration of Curcumin (Proteasome Inhibitor) or AG490 (JAK inhibitor) Reverses These Effects

TNF-*α* together with interferons attenuated the expression of MyHC IIa protein. On this evidence, we queried whether it is associated with lower activity of the respective gene (*Myhc IIa).* Surprisingly, TNF-*α*, IFN*α*, and IFN*γ* were shown to stimulate the activity of *Myhc IIa* by 3-, 1.8-, and 2.6-fold, respectively (Figure 7(b), *P* < 0.001 versus control). However, the *Myhc IIa* response to TNF-*α* was controlled by NF-*κ*B and STAT-1*α* transcription factors since the blockage of activation by curcumin (NF-*κ*B) or AG490 (STAT-1*α*) diminished TNF-*α*-dependent rise in *Myhc IIa* activity (Figures 7(c) and 7(d), *P* > 0.05 versus control). In contrast to TNF-*α*, curcumin did not affect the activity of *Myhc IIa* upon cotreatment with IFN*α* (Figure 7(c), *P* > 0.05 versus TNF-*α* and IFN*α*) but it increased *Myhc IIa* activity when TNF-*α* was added along with IFN*γ* (Figure 7(c), *P* < 0.01 versus TNF-*α* and IFN*γ*). Astonishingly, AG490 co-treatment with IFN*α* elevated *Myhc IIa* activity by 2.5-fold (Figure 7(d), *P* < 0.001 versus control).

### 3.13. TNF-*α* and IFN*α* Both Stimulate *Atrogin1* Gene Activity. TNF-*α*-Dependent Activation is Controlled by NF-*κ*B and STAT-1*α* as the Administration of Curcumin (Proteasome Inhibitor) or AG490 (JAK Inhibitor) Reversed This Effect: AG-490 Could Not Stop IFN*α*-Dependent *Atrogin1* Gene Activation

**Figure 8:**
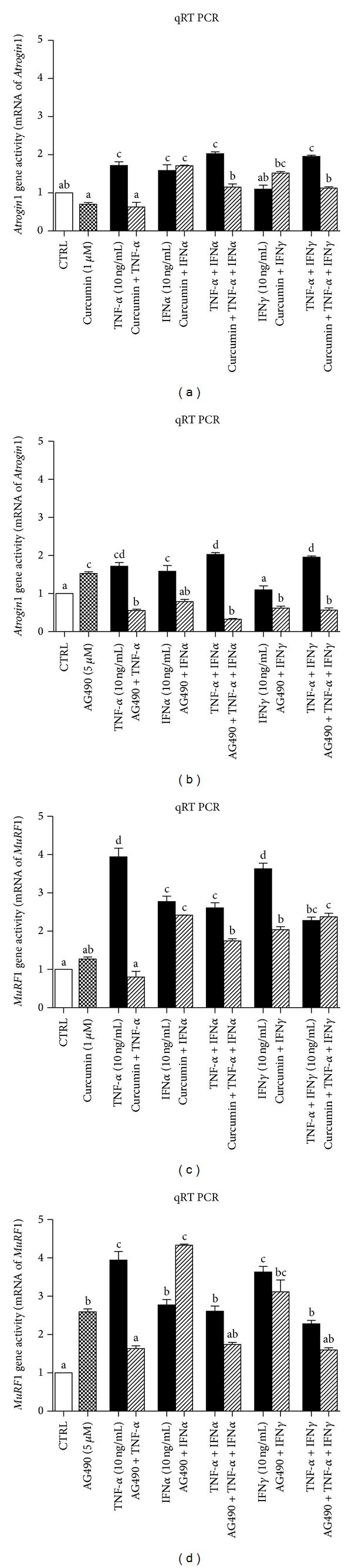
Long term effects of TNF-*α*, IFN*α*, and IFN*γ* given alone or together (10 ng/mL each, filled bars) or with metabolic inhibitors (slashed bars) at concentrations indicated on the *Atrogin1* and *MuRF1* gene activities in the 8th day of myogenesis. Fold increase was calculated according to the formula described in Section 2.

When TNF-*α* or IFN*α*, or both (10 ng/mL) were added to differentiating medium for the last 48 hours of differentiation (days 6–8), *Atrogin1* gene activity was stimulated by 1.8- and 1.6-fold at the 8th day of study (Figure 8(a), *P* < 0.01 compared to control). TNF-*α*, but not IFN*α*, dependent *Atrogin1* activation, was inhibited by curcumin (1 *μ*M, Figure 8(a), *P* > 0.05 versus control). A more pronounced effect was induced by AG490 (5 *μ*M), which almost completely abolished TNF-*α* and IFN*α* effects during the last 48 hours of the 8-day incubation time (Figure 8(b), *P* < 0.001 compared to control).

### 3.14. TNF-*α*, IFN*α*, and IFN*γ* Stimulate *MuRF1* Gene Activity: TNF-*α*- and IFN*γ*- but Not IFN*α*-Dependent Activation Are Controlled by NF-*κ*B and STAT-1*α* as the Administration of Curcumin (Proteasome Inhibitor) or AG490 (JAK Inhibitor) Reverses This Effect: AD-490 Further Stimulates IFN*α*-Dependent *MuRF1* Gene Activation

When TNF-*α*, IFN*α*, or IFN*γ* (10 ng/mL) were added to differentiating medium for the last 48 hours *MuRF1* gene activity was stimulated almost 4-fold at the 8th day of study (Figure 8(c), *P* < 0.001 compared to control). TNF-*α* and IFN*γ*, but not the IFN*α*-stimulated *MuRF1* gene activity in NF-*κ*B-dependent manner, since curcumin (1 *μ*M) was capable to reduce significantly the cellular response (Figure 8(d), *P* < 0.01 versus TNF-*α* and IFN*γ* alone). No effect of curcumin was observed with respect to IFN*α* (Figure 8(c), *P* > 0.05 versus IFN*α* alone). AG490 (5 *μ*M) abolished TNF-*α*-induced effect, but it potentiated the IFN*α* effect on *MuRF1* gene activity by almost 1.6-fold (Figure 8(d), *P* < 0.001 versus IFN*α* alone), whereas it had no significant influence on IFN*γ*-dependent stimulation of *MuRF1* gene activity (Figure 8(d), *P* > 0.05 versus IFN*γ* alone).

## 4. Discussion

Several signaling pathways have been shown to participate in cancer cachexia [25, 26]. It was reported that TGF-*β* superfamily (TGF-*β*, activin, GDF-15, and myostatin) contributes to muscle wasting through SMAD pathway activation [27], whereas blockage of actRIIB partially reversed this effect [28]. Also JAK/STAT3 pathway inhibition was demonstrated to improve IL-6-induced skeletal muscle cachexia in animal and C2C12 muscle cell models [13, 15]. Recently, we reported that leptin acts as mitogen but it markedly impairs muscle cell viability and myogenic differentiation through JAK/STAT3 signaling pathway [29]. As leptin deficient obese mice (ob^−^/ob^−^) have also reduced lean body mass, apparently other than leptin-dependent signaling pathways must be engaged in muscle cachexia. In this study we showed that TNF-*α* stimulated DNA synthesis similarly to leptin (Figure 1(c), *P* < 0.001 versus control) but it also increased C2C12 muscle cell viability (Figures 1(a)-1(b), *P* < 0.001 versus control). Thus, both cytokines seem to actively participate in the activation of satellite cells (myoblasts) as was previously reported for leukemia inhibitory factor LIF [30]. This effect of cytokine does not necessarily mean that muscle tissue would grow in hyperplasia-associated hypertrophy conditions, as the key event in muscle growth is differentiation and fusion. Moreover, mitogenicity has nothing to do with muscle fiber decay and resulting muscle cachexia. Among inflammatory cytokines, TNF-*α*/cachectin, IL-1*α*, IL-1*β*, IFN*γ*, and IL-6 have been implicated in muscle wasting, and their elevated levels were detected in cachectic patients [31]. In this experiment we sought to mimic the effects of increased concentrations of TNF-*α*, IFN*α*, and IFN*γ* during muscle differentiation using C2C12 mouse myoblasts. We also showed their respective effects in fully formed C2C12 myotubes. As TNF-*α* improved viability and mitogenicity of differentiating C2C12 myoblasts, we suggest that TNF-*α* might be capable to induce muscle dedifferentiation as demonstrated by Buck and Chojkier [32]. TNF-*α* improved cell respiration through NF-*κ*B-dependent mechanism, as curcumin (1 *μ*M) administered with TNF-*α* impaired muscle cell viability even stronger than curcumin given alone (Figure 1(a), *P* < 0.01). Inhibition of STAT-1*α* kinase JAK with tyrphostin (AG490) also retarded TNF-*α*-induced mitogenicity and viability below control level (Figures 1(b)-1(c), *P* < 0.001 versus control). A similar effect of AG490 on C2C12 myoblast proliferation was observed by Spangenburg and Booth [30]. Moreover, NF-*κ*B and STAT-1*α* responded to TNF-*α* in elevated transcriptional activity, as shown in the 3-day myotubes in short- and long term experiments (Figures 1(d)-1(e), 3(b), *P* < 0.05 versus control). These preliminary observations prompted us to study NF-*κ*B and STAT-1*α* effects in detail. Western blots confirmed transcriptional activation in the 3rd day of myogenesis as it was accompanied by higher nuclear NF-*κ*B and lower cytosolic I*κ*B protein levels after TNF-*α* (Figure 1(f)). Insulin pretreatment amplified TNF-*α*-dependent activity of NF-*κ*B (Figure 1(h), *P* < 0.001 versus TNF-*α* given alone), but it could hardly affect IFN*α* or IFN*γ* effects (Figure 1(i), *P* > 0.05). Insulin however, after initial pretreatment, given in combined treatment with TNF-*α*, inhibited nuclear translocation of NF-*κ*B (Figure 1(g)). TNF-*α* administration retarded muscle differentiation, as evidenced by lower MRFs (MyoD and myogenin) expression and reduced myotube formation (Figure 6(a) and supplementary data 3). It is well established that TNF-*α* controls NF-*κ*B activity in muscle cells [5, 32] and that transcriptional regulation through NF-*κ*B controls MyoD decay in C2C12 myotubes [5, 33]. NF-*κ*B regulates the expression of a variety of muscle genes and proteins including those involved in control of cell proliferation [34], myogenesis, oxidative stress, and mitochondrial dysfunction [35]. Thus, higher NF-*κ*B activity observed after TNF-*α* administration in this experiment suggests that differentiating myoblasts could be withdrawn from the myogenic program as shown recently in muscle-derived stem cells isolated from transgenic mice [36], or by enforced expression of c-FLIP in satellite cells [37]. Moreover, c-FLIP is a known intracellular modulator of death receptor mediated signaling (inhibition of intrinsic apoptosis) and NF-*κ*B activator [38]. Actually, this was not the case for IFN*α* and IFN*γ* as neither cytokine (10 ng/mL) could affect NF-*κ*B activity (Figure 1(e), *P* > 0.05 versus control). However, IFN*α* and IFN*γ* used in one-day pretreatment sensitized C2C12 myotubes to TNF-*α*-induced NF-*κ*B activation on the 3rd day of myogenesis (Figure 2(b), *P* < 0.001). Higher NF-*κ*B transcriptional activity upon pretreatment with IFNs was confirmed by higher and earlier nuclear expression of NF-*κ*B (Figure 2(a)). This observation points to interferons as competent cytokines capable to amplify TNF-*α*-induced inflammatory response. Indeed, systemic inflammation was often indicated as causal to skeletal muscle wasting [39, 40]. In this study, we successfully verified that IFN*α* and IFN*γ* affected TNF-*α*-dependent NF-*κ*B activation and showed that the latter occured through STAT-1*α* activation. As shown in Figure 2(f) active STAT-1*α* (phosphorylated at tyrosine 701, STAT-1*α*-P-Y^701^) has been detected in the nucleus after TNF-*α*, IFN*α*, and IFN*γ*, whereas insulin pre- and cotreatment could not reduce the effect of IFNs. Thus, it is apparent that TNF-*α*, IFN*α*, and IFN*γ* cooperate in NF-*κ*B activation, and that nonactive STAT-1*α* seems to inhibit this process. Next, we assumed that STAT-1*α* might inhibit NF-*κ*B in nonstimulated muscle cells as STAT-1*α* was previously reported in other cell-types to be assembled with TNF-R1 through TRADD protein partner [41–43]. Actually, during the immunoprecipitation study IFN*α* and IFN*γ* caused reduction in the STAT-1*α* protein quantity bound to TRADD protein (important component of TNF-*α*-induced signalosome, Figure 7(a)). Thus, withdrawal of STAT-1*α* from TNF-R1 assembly by IFN*α* or IFN*γ* might explain their amplifying effect of TNF-*α*-induced NF-*κ*B activation. Similarly, IFN mediated release from STAT-1*α*-mediated inhibition of NF-*κ*B activation in human colon adenocarcinoma COLO 205 cells [44]. Raising evidence obtained from similar experiments performed in the past propose phylogenetically conserved mechanism of STAT-1*α*-dependent inhibition of NF-*κ*B. Insulin, which is known to activate NF-*κ*B through PI3-K/Akt pathway [45], was also capable to sensitize C2C12 myotubes to TNF-*α*-dependent NF-*κ*B activation (Figure 1(h), *P* < 0.001 versus control), but it did not change the response of NF-*κ*B or STAT-1*α* to interferons (Figures 1(i) and 2(g), *P* > 0.05 versus control). Therefore, we assume that similarities between insulin and interferons in TNF-*α*-dependent NF-*κ*B activation must have different origins. Insulin was capable to reduce NF-*κ*B activation in the presence of IFN*α*- but not IFN*γ* (Figures 2(d)-2(e)), although insulin did not influence nuclear NF-*κ*B and cytosolic I*κ*B protein levels, respectively, (Figure 2(c)). Presumably, the effects of insulin on TNF-*α*- and IFNs-mediated NF-*κ*B activation were indirect and not related to STAT-1*α* activity (Figure 2(b), *P* > 0.05 versus TNF-*α* or IFNs). Interestingly, TNF-*α* raised nuclear STAT-1*α*-P-Y^701^ protein levels, while insulin pre- and co-treatment additionally amplified this effect in the 3rd day of myogenic differentiation (Figure 3(a)). At the same time, TNF-*α* evoked higher transcriptional activity of STAT-1*α* (Figure 3(b), *P* < 0.05). These results are in concert with our previous report [45] as IFN*γ* did not (Figure 3(b), *P* > 0.05) but IFN*α* did increase TNF-*α*-dependent STAT-1*α* activation (Figure 3(d), *P* < 0.05). Insulin pretreatment did not affect IFN*α*-induced, TNF-*α*-dependent activation of STAT-1*α* (Figure 3(d), *P* < 0.05). The latter observation was corroborated by WB results obtained for STAT-1*α*-P-Y^701^ protein levels (Figures 3(c) and 3(e)). One should keep in mind, that multiple signaling pathways are relevant to muscle cachexia (NF-*κ*B, JAK/STAT, SMAD). From a pathophysiological point of view, muscle cachexia is an adaptation mechanism to chronic inflammation and long lasting anorexia (cancer, anorexia nervosa, and AIDS). It has pushed through the demands for amino-acids needed to meet the requirements of acute phase protein synthesis. Interestingly, it is clear now that skeletal muscles not only play a pivotal role in mobilization of amino-acids, but they are also the site of extensive production of acute phase proteins [13]. Overall, the goal is not to ultimately stop muscle cachexia by pharmacological or biotechnological intervention, but to make this task obsolete through the correction of associated symptoms of disease.

The outcome of MyHC IIa protein expression in 6-day myotubes challenged with cytokines was chosen to examine their procachectic activity. Initially, it should be stressed that muscle differentiation of C2C12 myoblasts was suppressed by TNF-*α* but not IFN*α* or IFN*γ*, except when they were administered together with TNF-*α* (Figure 6(a)). These effects were partially under the control of NF-*κ*B and/or JAK/STAT-1*α*, since either curcumin (NF-*κ*B inhibitor) or AG490 (JAK inhibitor) reversed some of the TNF-*α*-induced effects. Curcumin is a dominant NF-*κ*B inhibitor as it prevents proteasomal degradation of I*κ*B and subsequent NF-*κ*B activation [46]. In 6-day myotubes, TNF-*α*, given alone or together with IFN*α* or IFN*γ* increased nuclear expression of NF-*κ*B with simultaneous drop in cytosolic expression of I*κ*B (Figure 5(a)). At the same time, TNF-*α* administered alone reduced the expression of the fast form of MyHC IIa protein (Figure 5(b)). These data suggest that NF-*κ*B is a negative regulator of myogenesis, and that NF-*κ*B delays the rise in MyHC IIa protein expression. Marked reduction was also observed after concomitant administration of IFN*α* and IFN*γ*. However, neither cytokine administered separately nor in used combinations could induce nuclear expression of NF-*κ*B upon cotreatment with curcumin (1 *μ*M), and associated cytosolic I*κ*B expression level remained high (Figure 5(a)). JAK inhibitor AG490 (5 *μ*M) was less potent than curcumin with respect to NF-*κ*B/I*κ*B expression levels (Figure 5(a)). From this study it becomes clear that myogenesis is repressed by NF-*κ*B as reported by Kumar et al. [47]. The reduced expression of fast form of MyHC IIa was observed after TNF-*α*, but not IFN*α* or IFN*γ* given alone (Figure 5(b)). Combined treatment of TNF-*α* and IFN*α* and IFN*α* and IFN*γ* was even more powerful to repress MyHC IIa expression in the cytosol. Both inhibitors (curcumin and AG490) almost totally abrogated the above-mentioned effects of cytokines (Figure 5(b)). It should be underlined that TNF-*α* markedly decreased the MyoD/myogenin protein levels (Figure 6) with resultant drop in MyHC IIa protein expression. Neither IFN*α* nor IFN*γ* could affect myogenin expression, but similarly to TNF-*α*, both interferons considerably diminished MyoD protein expression levels (Figure 6). Summing up, combined treatment of TNF-*α* with IFN*α* or IFN*γ* led to a significant effect evoked by the former (Figure 6). Thus, in this experiment TNF-*α* seems to play an important negative role in the control of myogenesis.

To make the picture of cytokine-dependent muscle differentiation and muscle fiber decay more lucid, additional experiments followed by qRT PCR analysis have been performed. Observations obtained from this study suggest marked differences in the control of muscle differentiation and muscle wasting at genomic and translational levels. *MyHC IIa* gene expression was raised noticeably after TNF-*α* administration (Figures 7(c)-7(d), *P* < 0.001 versus control), which was unexpected as MyoD level was diminished at the same time. Interferons also stimulated *MyHC IIa* gene expression, but they were apparently weaker (Figures 7(c)-7(d), *P* < 0.001 versus control). Astonishingly, curcumin (1 *μ*M) was unable to cease the action of either cytokine (Figure 7(c), *P* > 0.05 versus cytokines alone), although it markedly reduced the effect of TNF-*α* and IFN*γ* (Figure 7(c), *P* < 0.001 versus cytokines alone). AG490 significantly elevated *MyHC IIa* gene expression either alone or when it was given together with interferons (Figure 7(d), *P* < 0.01 versus control), but it abrogated TNF-*α*- but not IFN*α*-induced *MyHC IIa* gene activation. As MyHC IIa protein level decreased upon TNF-*α* or TNF-*α* and IFNs administration, two genes *Atrogin1* and *MuRF1* encoding respective ubiquitin ligases were examined. These genes were previously indicated as crucial for muscle fiber decay [48, 49]. TNF-*α* and IFN*γ* stimulated (Figure 8(a), *P* < 0.001 versus control), whereas IFN*α* did not affect *Atrogin1* gene expression (Figure 8(a), *P* > 0.05 versus control). Curcumin protected myotubes from TNF-*α*-, TNF-*α* and IFNs-stimulated* Atrogin1* gene expression (Figure 8(a), *P* > 0.05 versus control). Cytokine-induced *Atrogin1* gene activation was considerably diminished by AG490; gene expression even dropped below the control level (Figure 8(b), *P* < 0.05 versus control). Surprisingly, AG490 given alone stimulated gene expression (Figure 8(b), *P* < 0.01 versus control).* MuRF1* gene expression was activated by cytokines in a similar manner (Figures 8(c)-8(d), *P* < 0.001 versus control) but curcumin and AG490 could repress only TNF-*α*-induced gene activation (Figures 8(c)-8(d), *P* < 0.001 versus TNF-*α* alone). These results are in agreement with the well known effects of transcriptional activity of NF-*κ*B, as both genes are targets in muscle wasting [19, 50]. Much less is known about the control of these genes by STAT-1*α*. In this study, however, AG490 drastically reduced *Atrogin1* gene expression when added with cytokines. AG490 used individually elevated activity of *Atrogin1* gene (Figure 8(b), *P* < 0.01 versus control). Monitoring of *Atrogin1* and *MuRF1* genes activity showed dominant effect of TNF-*α* (also in combined treatment with IFNs). We speculate that NF-*κ*B was a major player at the genomic level for TNF-*α*-induced effects. Where TNF-*α* was administered together with IFNs, curcumin addition could not repress increased *Atrogin1* and *MuRF1* genes activity (Figures 8(a) and 8(c), *P* > 0.05 versus TNF-*α* and IFNs). A similar effect was noted in the case of treatment with AG490. As mentioned above, this JAK/STAT inhibitor normalized *Atrogin1* gene activity which was raised by TNF-*α* and IFNs, but it did not affect *MuRF1* gene activity except for the situation when TNF-*α* was given alone (Figure 8(d), *P* < 0.001 versus control).

## 5. Conclusions

Our hypothesis addresses competition between cytokines (TNF-*α*, IFN*α*, IFN*γ*) to recruit STAT-1*α*. During this process release of NF-*κ*B allowed both NF-*κ*B and STAT-1*α* to cooperate at the genomic level. As we showed, some of the cachectic effects were evoked differently for TNF-*α*, IFNs, and both, as TNF-*α*-induced activation of *Atrogin1* and *MuRF1* genes was reduced by NF-*κ*B and STAT-1*α* inhibitors (curcumin and AG490, resp.), while the presence of IFNs deteriorated curcumin effect. Astonishingly, TNF-*α* was apparently mitogenic and stimulated viability of mononuclear muscle cells, whereas this cytokine efficiently inhibited muscle fiber formation. Moreover, TNF-*α* indirectly and directly diminished MyHC IIa protein level, even though it stimulated *MyHC IIa* gene expression. From qRT PCR study it became obvious that proteasome-ubiquitin system activity was activated. Actually, such reaction was previously reported by Tan et al. [51]. Similarly to *Atrogin1* and *MuRF1*, the activation of *MyHC IIa* gene by TNF-*α* could be partially reversed by NF-*κ*B and STAT-1*α* inhibitors. The effect of NF-*κ*B and STAT-1*α* inhibitors was diminished upon IFNs cotreatment pointing to the synergy between TNF-*α* and IFNs in action. Insulin did not prevent combined TNF-*α*- and IFN-s-dependent muscle decay, even though it is almost ultimate anabolic hormone. Thus, where anyone thought to bring to an end the TNF-*α*-induced muscle cachexia, either the secretion of this cytokine or IFNs seems to be essential [52].

## Supplementary Material

Supplementary material 1: Dose-response and time-dependent curves showing long term effects of TNF-*α*, IFN*α*, IFN*γ* (10 ng/mL each) on cell viability.
Supplementary material 2: Immunofluorescent detection of NF-*κ*B location in 3-day old C2C12 myotubes using confocal microscopy.
Supplementary material 3: Myotube formation from C2C12 myoblasts.

## Figures and Tables

**Table 1:** 

Gene	Primers and their sequences	MgCl_2_ (mM)	Product (bp)
*Myhc IIa *	F: 5′-GGAGAAGAGCGAGCTGAAGA-3′ R: 5′-GGAAAACTCGCCTGACTCTG-3′	3	208
*Atrogin1 *	F: 5′-CTCTGTACCATGCCGTTCCT-3′ R: 5′-GGCTGCTGAACAGATTCTCC-3′	3	179
*MuRF1 *	F: 5′-GACAGTCGCATTTCAAAGCA-3′ R: 5′-AACGACCTCCAGACATGGAC-3′	3	239
*18S rRNA *	F: 5′-GGAGAGCGGGTAAGAGAGGT-3′ R: 5′-CAGGACTAGGCGGAACAGAG-3′	2	235

## References

[1] Evans WJ, Morley JE, Argilés J (2008). Cachexia: a new definition. *Clinical Nutrition*.

[2] Tisdale MJ (2009). Mechanisms of cancer cachexia. *Physiological Reviews*.

[3] Hasselgren P, Fischer JE (2001). Muscle cachexia: current concepts of intracellular mechanisms and molecular regulation. *Annals of Surgery*.

[4] Cheshire JL, Baldwin AS (1997). Synergistic activation of NF-*κ*B by tumor necrosis factor alpha and gamma interferon via enhanced I*κ*B*α* degradation and de novo I*κ*B*β* degradation. *Molecular and Cellular Biology*.

[5] Guttridge DC, Mayo MW, Madrid LV, Wang C-Y, Baldwin A.S. J (2000). NF-*κ*B-induced loss of MyoD messenger RNA: possible role in muscle decay and cachexia. *Science*.

[6] Acharyya S, Ladner KJ, Nelsen LL (2004). Cancer cachexia is regulated by selective targeting of skeletal muscle gene products. *The Journal of Clinical Investigation*.

[7] Wheeler MT, Snyder EC, Patterson MN, Swoap SJ (1999). An E-box within the MHC IIB gene is bound by MyoD and is required for gene expression in fast muscle. *American Journal of Physiology—Cell Physiology*.

[8] Dehoux M, Gobier C, Lause P, Bertrand L, Ketelslegers J, Thissen J (2007). IGF-I does not prevent myotube atrophy caused by proinflammatory cytokines despite activation of Akt/Foxo and GSK-3*β* pathways and inhibition of atrogin-1 mRNA. *American Journal of Physiology—Endocrinology and Metabolism*.

[9] Cong H, Sun L, Liu C, Tien P (2011). Inhibition of atrogin-1/MAFbx expression by adenovirus-delivered small hairpin RNAs attenuates muscle atrophy in fasting mice. *Human Gene Therapy*.

[10] Ebert SM, Dyle MC, Kunkel SD (2012). Stress-induced skeletal muscle Gadd45a expression reprograms myonuclei and causes muscle atrophy. *The Journal of Biological Chemistry*.

[11] Fanzani A, Conraads VM, Penna F, Martinet W (2012). Molecular and cellular mechanisms of skeletal muscle atrophy: an update. *Journal of Cachexia, Sarcopenia and Muscle*.

[12] Sakuma K, Yamaguchi A (2012). Sarcopenia and cachexia: the adaptations of negative regulators of skeletal muscle mass. *Journal of Cachexia, Sarcopenia and Muscle*.

[13] Bonetto A, Aydogdu T, Jin X (2011). JAK/STAT3 pathway inhibition blocks skeletal muscle wasting downstream of IL-6 and in experimental cancer cachexia. *American Journal of Physiology—Endocrinology and Metabolism*.

[14] Bonetto A, Aydogdu T, Kunzevitzky N (2012). STAT3 activation in skeletal muscle links muscle wasting and the acute phase response in cancer cachexia. *PLoS ONE*.

[15] De Larichaudy J, Zufferli A, Serra F (2012). TNF-*α*- and tumor-induced skeletal muscle atrophy involves sphingolipid metabolism. *Skeletal Muscle*.

[16] Melstrom LG, Melstrom KA, Ding X-Z, Adrian TE (2007). Mechanisms of skeletal muscle degradation and its therapy in cancer cachexia. *Histology and Histopathology*.

[17] Zhou X, Wang JL, Lu J (2011). Reversal of cancer cachexia and muscle wasting by ActRIIB antagonism leads to prolonged survival. *Cell*.

[18] Karin M, Lin A (2002). NF-*κ*B at the crossroads of life and death. *Nature Immunology*.

[19] Cai D, Frantz JD, Tawa NE (2004). IKK*β*/NF-*κ*B activation causes severe muscle wasting in mice. *Cell*.

[20] Mourkioti F, Rosenthal N (2008). NF-*κ*B signaling in skeletal muscle: prospects for intervention in muscle diseases. *Journal of Molecular Medicine*.

[21] Reed SA, Senf SM, Cornwell EW, Kandarian SC, Judge AR (2011). Inhibition of IkappaB kinase alpha (IKK*α*) or IKKbeta (IKK*β*) plus forkhead box O (Foxo) abolishes skeletal muscle atrophy. *Biochemical and Biophysical Research Communications*.

[22] Yaffe D, Saxel O (1977). Serial passaging and differentiation of myogenic cells isolated from dystrophic mouse muscle. *Nature*.

[23] Jacobson MD, Burne JF, Raff MC (1994). Programmed cell death and Bcl-2 protection in the absence of a nucleus. *The EMBO Journal*.

[24] Bradford MM (1976). A rapid and sensitive method for the quantitation of microgram quantities of protein utilizing the principle of protein dye binding. *Analytical Biochemistry*.

[25] Argilés JM, Busquets S, Toledo M, López-Soriano FJ (2009). The role of cytokines in cancer cachexia. *Current Opinion in Supportive and Palliative Care*.

[26] Morley JE, Thomas DR, Wilson MG (2006). Cachexia: pathophysiology and clinical relevance. *American Journal of Clinical Nutrition*.

[27] Zimmers TA, Davies MV, Koniaris LG (2002). Induction of cachexia in mice by systemically administered myostatin. *Science*.

[28] Busquets S, Toledo M, Orpí M (2012). Myostatin blockage using actRIIB antagonism in mice bearing the Lewis lung carcinoma results in the improvement of muscle wasting and physical performance. *Journal of Cachexia, Sarcopenia and Muscle*.

[29] Pijet M, Pijet B, Litwiniuk A, Pająk B, Gajkowska B, Orzechowski A (2013). Leptin impairs myogenesis in C2C12 cells through JAK/STAT and MEK signaling pathways. *Cytokine*.

[30] Spangenburg EE, Booth FW (2002). Multiple signaling pathways mediate LIF-induced skeletal muscle satellite cell proliferation. *American Journal of Physiology—Cell Physiology*.

[31] Argiles JM, Bosquets S, Lopez-Soriano FJ (2003). Cytokines in the pathogenesis of cancer cachexia. *Current Opinion in Clinical Nutrition and Metabolic Care*.

[32] Buck M, Chojkier M (1996). Muscle wasting and dedifferentiation induced by oxidative stress in a murine model of cachexia is prevented by inhibitors of nitric oxide synthesis and antioxidants. *The EMBO Journal*.

[33] Di Marco S, Mazroui R, Dallaire P (2005). NF-*κ*B-mediated MyoD decay during muscle wasting requires nitric oxide synthase mRNA stabilization, HuR protein, and nitric oxide release. *Molecular and Cellular Biology*.

[34] Guttridge DC, Albanese C, Reuther JY, Pestell RG, Baldwin AS (1999). NF-*κ*B controls cell growth and differentiation through transcriptional regulation of cyclin D1. *Molecular and Cellular Biology*.

[35] Bhatnagar S, Panguluri SK, Gupta SK, Dahiya S, Lundy RF, Kumar A (2010). Tumor necrosis factor-*α* regulates distinct molecular pathways and gene networks in cultured skeletal muscle cells. *PLoS ONE*.

[36] Lu A, Proto JD, Guo L (2012). NF-*κ*B negatively impacts the myogenic potential of muscle-derived stem cells. *Molecular Therapy*.

[37] Giampietri C, Petrungaro S, Coluccia P (2010). c-flip overexpression affects satellite cell proliferation and promotes skeletal muscle aging. *Cell Death and Disease*.

[38] Kataoka T, Budd RC, Holler N (2000). The caspase-8 inhibitor FLIP promotes activation of NF-*κ*B and Erk signaling pathways. *Current Biology*.

[39] Dunlop RJ, Campbell CW (2000). Cytokines and advanced cancer. *Journal of Pain and Symptom Management*.

[40] Jackman RW, Kandarian SC (2004). The molecular basis of skeletal muscle atrophy. *American Journal of Physiology—Cell Physiology*.

[41] Wang Y, Wu TR, Cai S, Welte T, Chin YE (2000). Stat1 as a component of tumor necrosis factor alpha receptor 1-TRADD signaling complex to inhibit NF-*κ*B activation. *Molecular and Cellular Biology*.

[42] Wesemann DR, Benveniste EN (2003). STAT-1*α* and IFN-*γ* as modulators of TNF-*α* signaling in macrophages: regulation and functional implications of the TNF receptor 1:STAT-1*α* complex. *Journal of Immunology*.

[43] Wesemann DR, Qin H, Kokorina N, Benveniste EN (2004). TRADD interacts with STAT1-*α* and influences interferon-*γ* signaling. *Nature Immunology*.

[44] Pajak B, Orzechowski A (2007). IFN-alpha competes with TNF-alpha for STAT-1alpha; molecular basis for immune escape of human colon adenocarcinoma COLO 205 cells. *Oncology Reports*.

[45] Mitsiades CS, Mitsiades N, Poulaki V (2002). Activation of NF-*Κ*B and upregulation of intracellular anti-apoptotic proteins via the IGF-1/Akt signaling in human multiple myeloma cells: therapeutic implications. *Oncogene*.

[46] Jobin C, Bradham CA, Russo MP (1999). Curcumin blocks cytokine-mediated NF-*κ*B activation and proinflammatory gene expression by inhibiting inhibitory factor I-*κ*B kinase activity. *Journal of Immunology*.

[47] Kumar NB, Kazi A, Smith T (2010). Cancer cachexia: traditional therapies and novel molecular mechanism-based approaches to treatment. *Current Treatment Options in Oncology*.

[48] Bodine SC, Latres E, Baumhueter S (2001). Identification of ubiquitin ligases required for skeletal Muscle Atrophy. *Science*.

[49] Gomes MD, Lecker SH, Jagoe RT, Navon A, Goldberg AL (2001). Atrogin-1, a muscle-specific F-box protein highly expressed during muscle atrophy. *Proceedings of the National Academy of Sciences of the United States of America*.

[50] Acharyya S, Guttridge DC (2007). Cancer cachexia signaling pathways continue to emerge yet much still points to the proteasome. *Clinical Cancer Research*.

[51] Tan Y, Peng X, Wang F, You Z, Dong Y, Wang S (2006). Effects of tumor necrosis factor-alpha on the 26S proteasome and 19S regulator in skeletal muscle of severely scalded mice. *Journal of Burn Care and Research*.

[52] Pajak B, Orzechowska S, Pijet B (2008). Crossroads of cytokine signaling: the chase to stop muscle cachexia. *Journal of Physiology and Pharmacology*.

