# Supplementary figures and images for: TNF-α and IFN-s-Dependent Muscle Decay Is Linked to NF-κB- and STAT-1α-Stimulated Atrogin1 and MuRF1 Genes in C2C12 Myotubes
Source: Mediators Inflamm. 2013 Dec 17;2013:171437. doi: 10.1155/2013/171437 (PMC3877628; doi:10.1155/2013/171437)

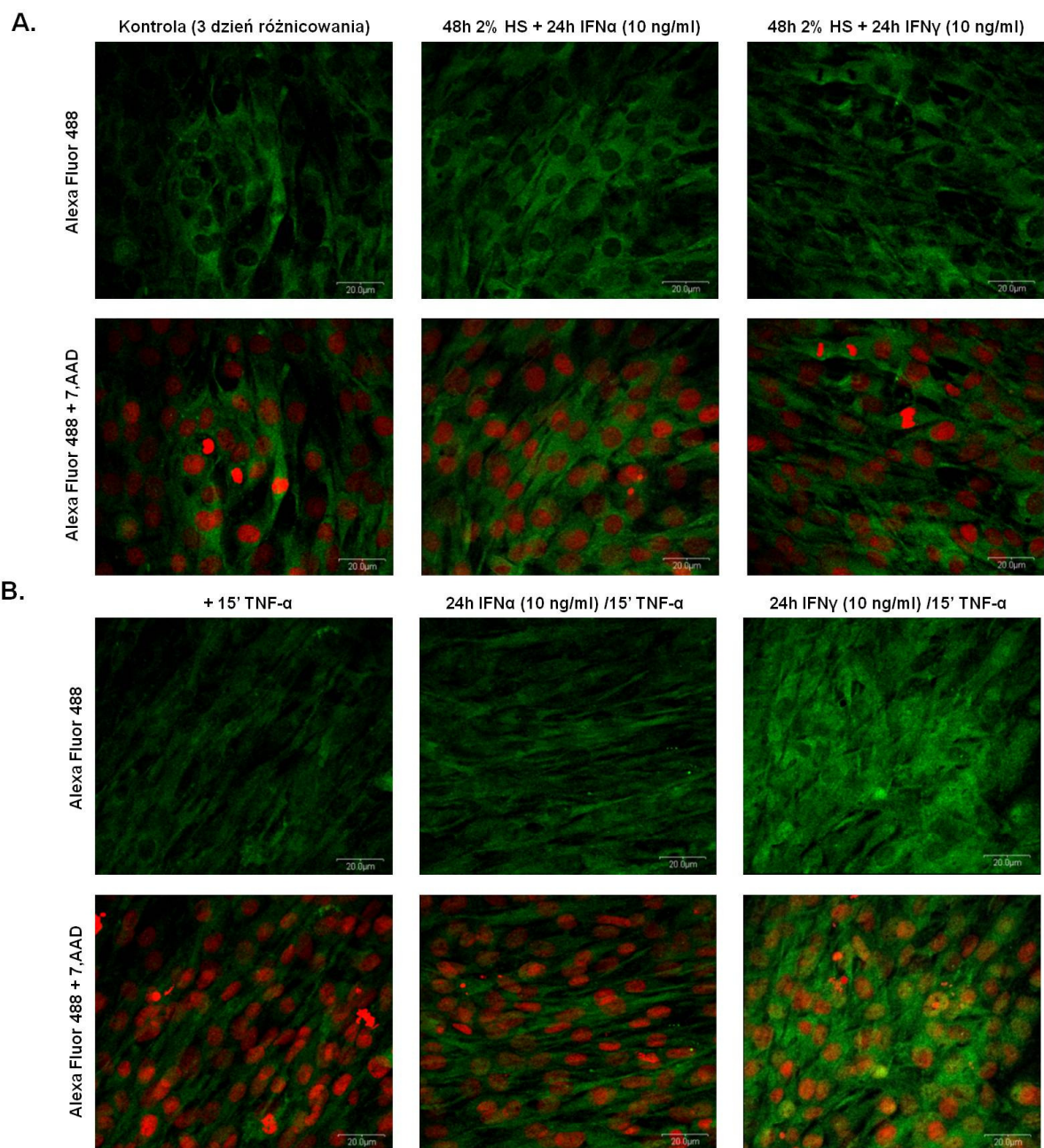

Supplement: Supplementary file 1 — Supplementary material 1: Dose-response and time-dependent curves showing long term effects of TNF-α, IFNα, IFNγ (10 ng/mL each) on cell viability. Supplementary material 2: Immunofluorescent detection of NF-κB location in 3-day old C2C12 myotubes using confocal microscopy. Supplementary material 3: Myotube formation from C2C12 myoblasts. [file 171437.f1.pdf]

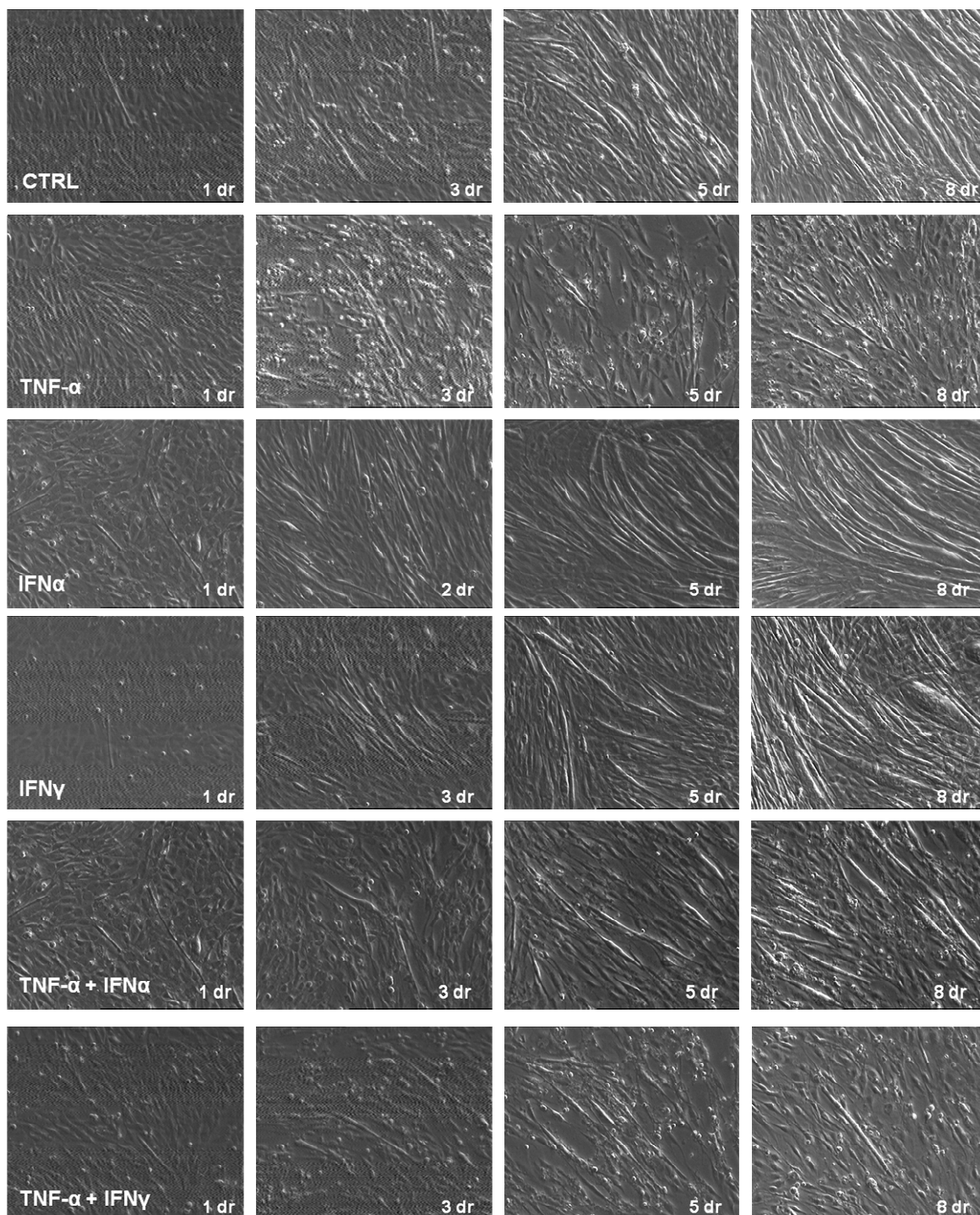

Supplement: Supplementary file 2 [file 171437.f2.pdf]

### A. MTT

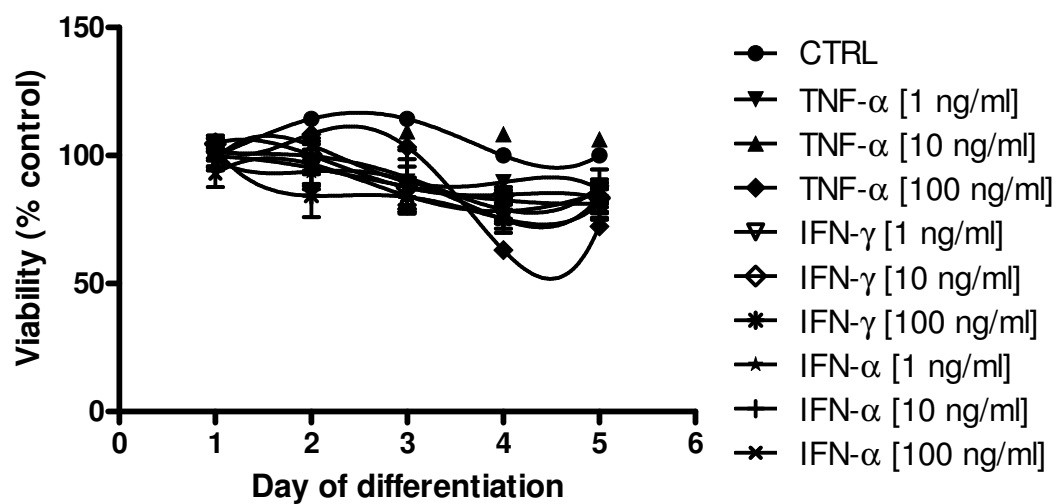

### B. MTT

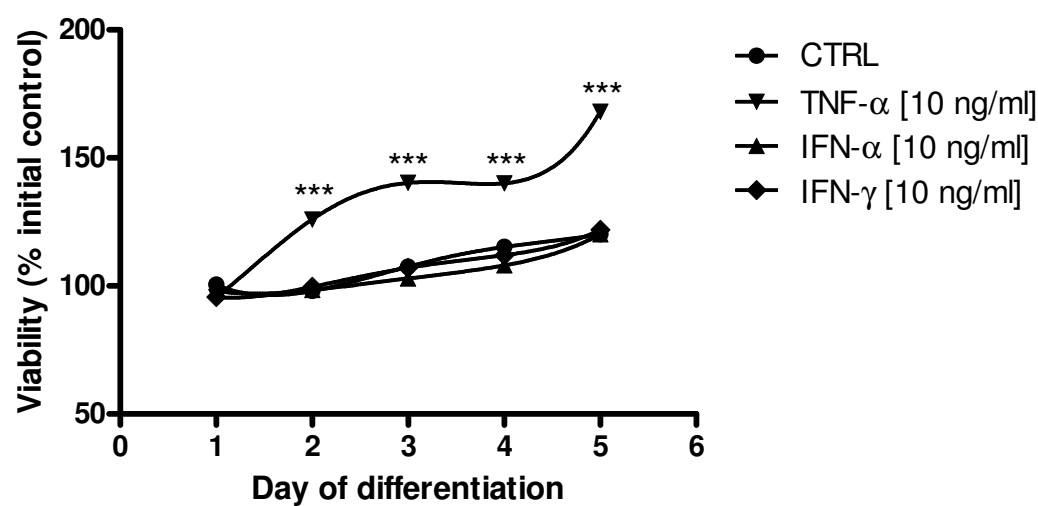

### C. MTT

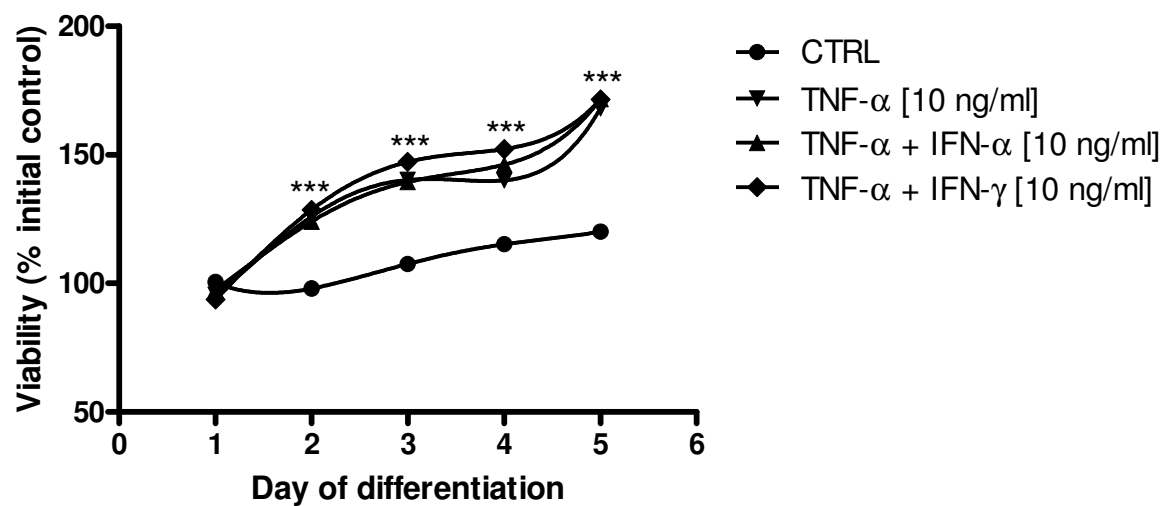

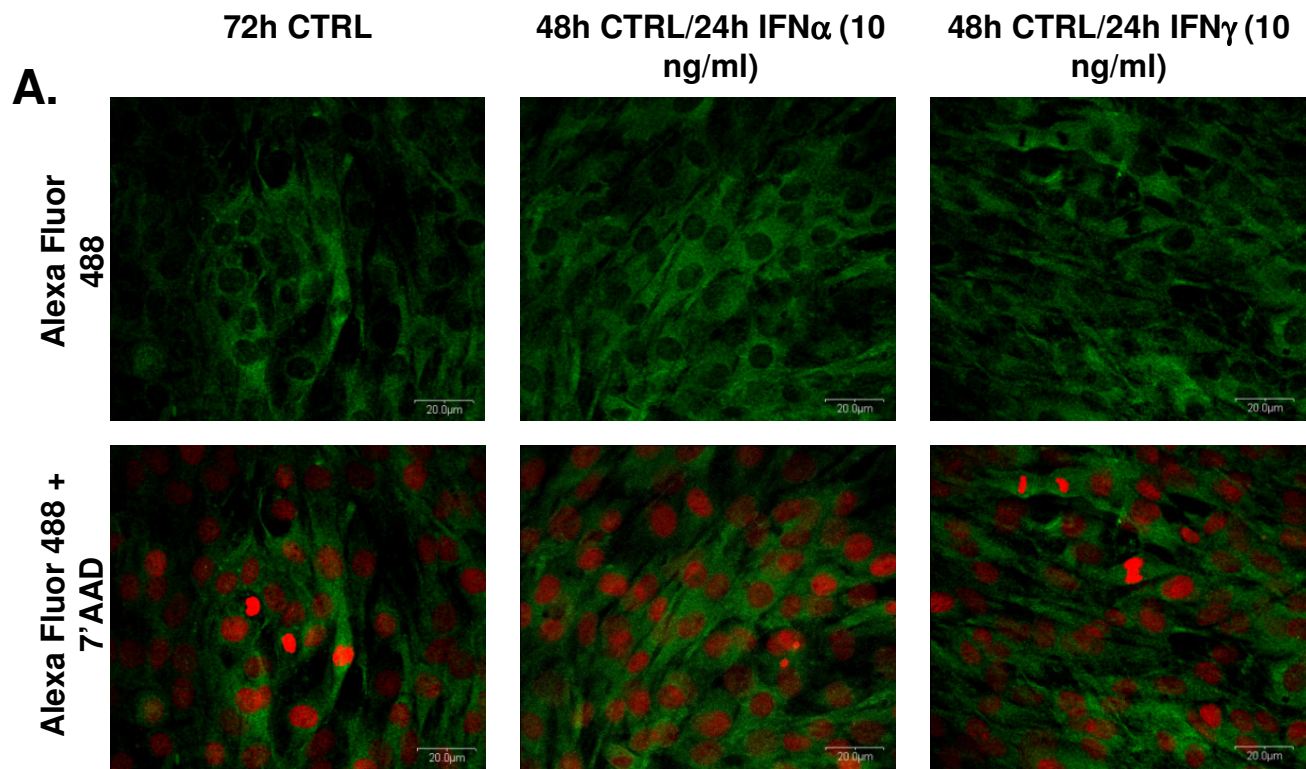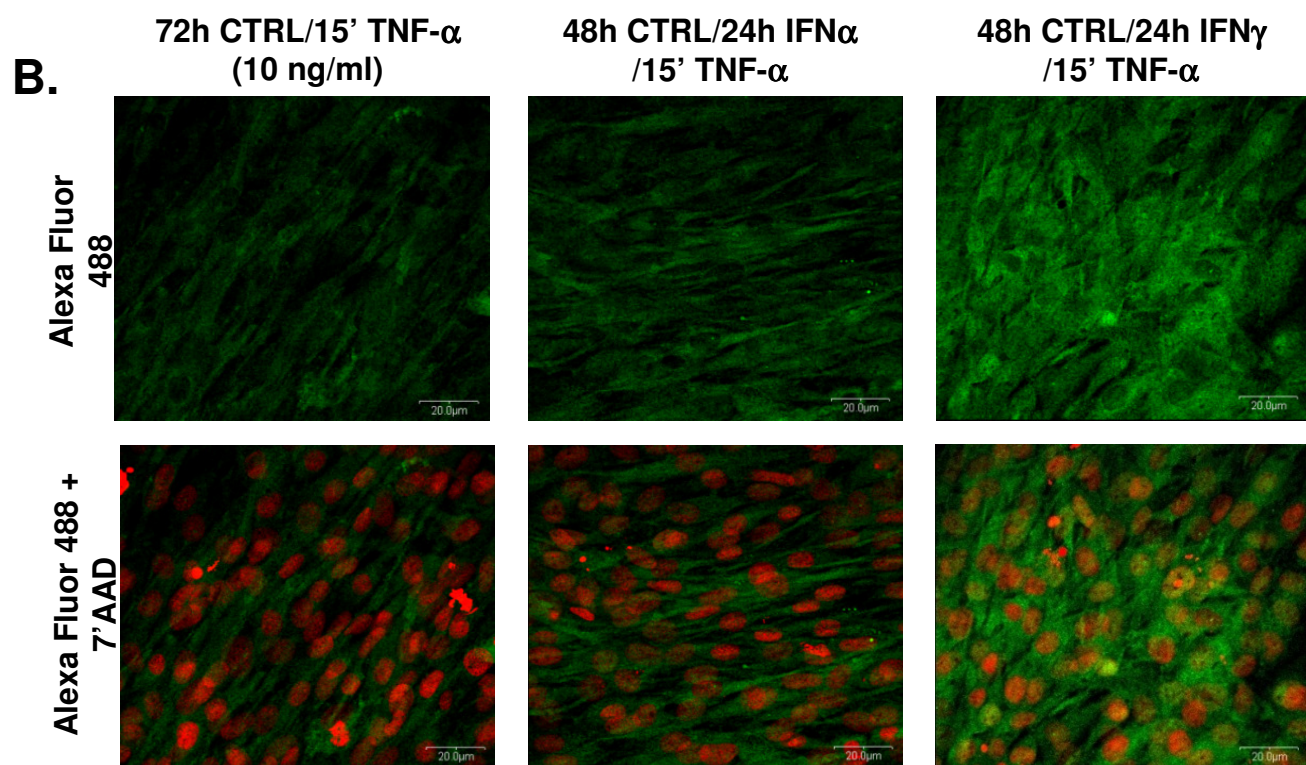

**C.**

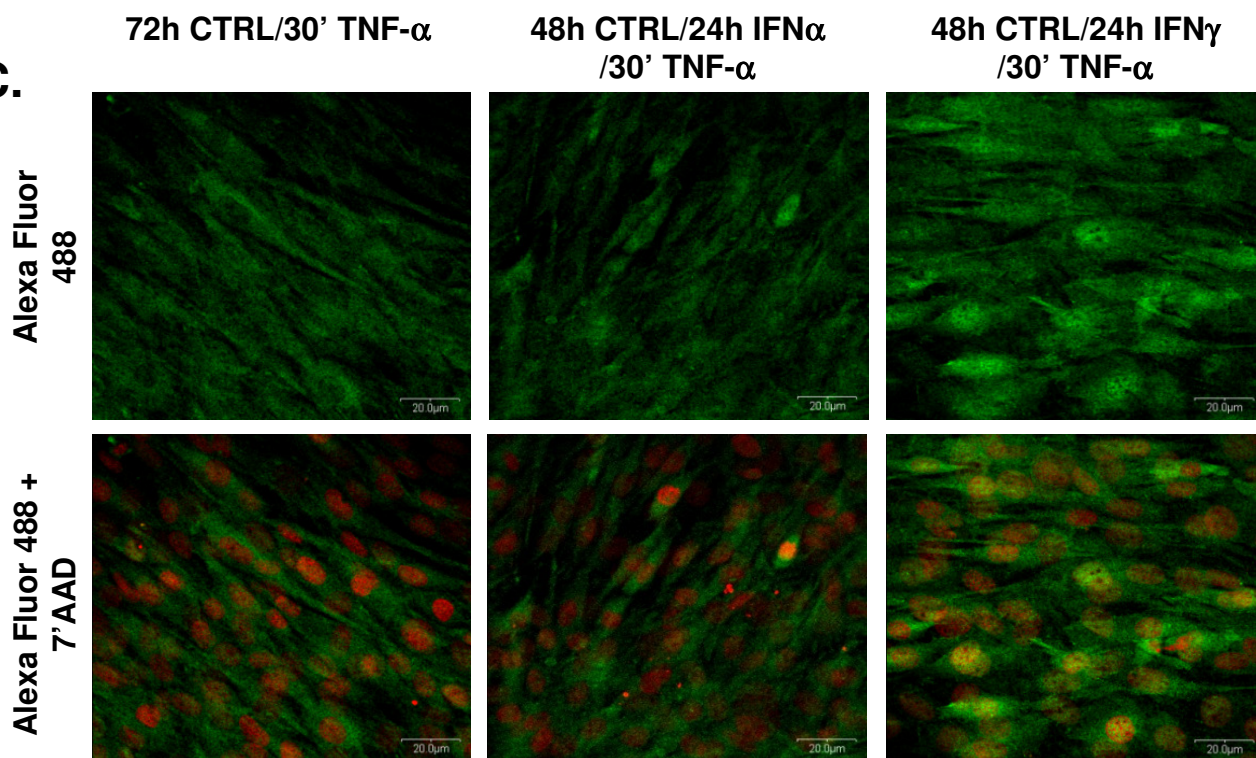

**D.**

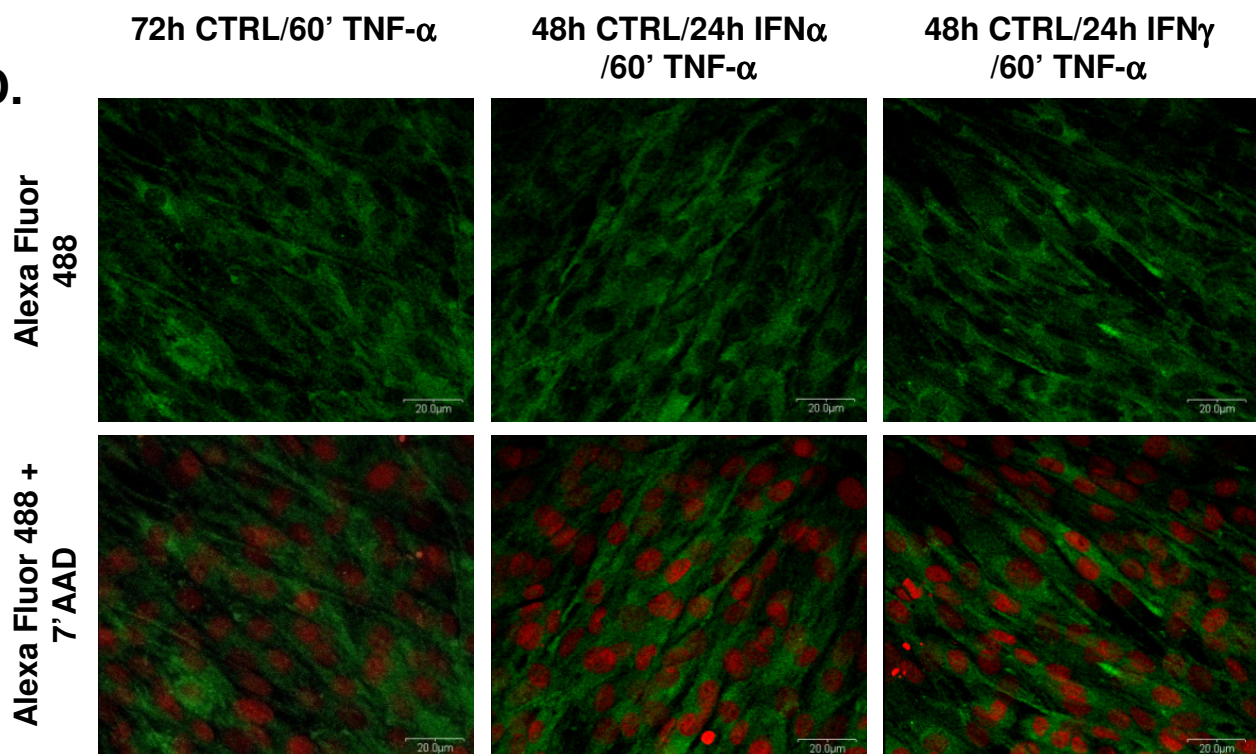

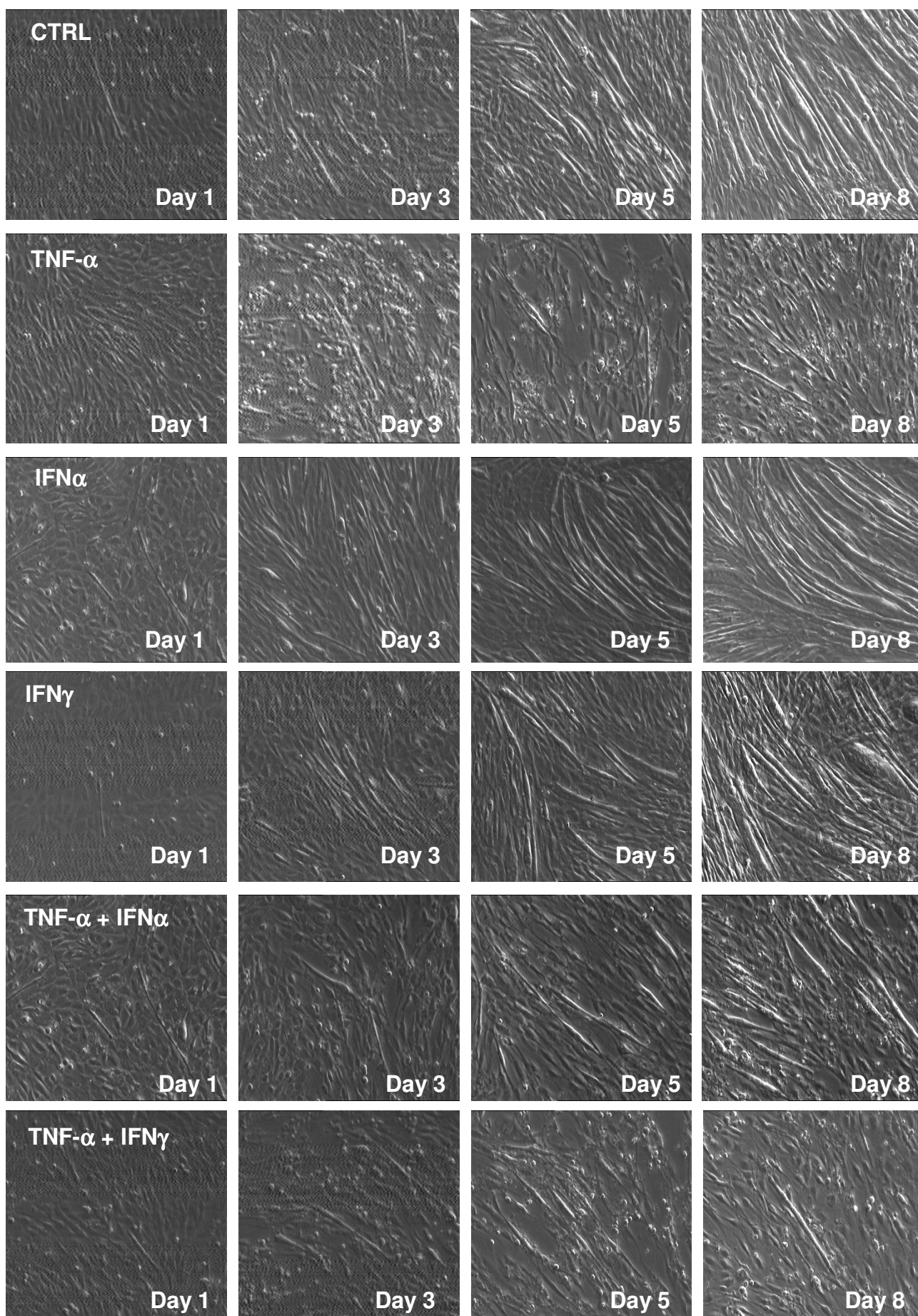

Supplement: Supplementary file 3 [file 171437.f3.pdf]
